# What a Difference a Water Molecule Makes—A Combined Experimental/Theoretical Study on 2,3,5-triphenyl-2*H*-tetrazol-3-ium Chloride Hydrate in Solution and the Solid-State

**DOI:** 10.3390/molecules31010138

**Published:** 2025-12-31

**Authors:** Rim Bechaieb, Maha F. El-Tohamy, Haitham AlRabiah, Gamal A. E. Mostafa, Bruno Poti e Silva, Maryam Niazi, Axel Klein

**Affiliations:** 1 Laboratoire de Chimie Théorique, Sorbonne Université UPMC, University Paris 06, UMR 7616, F-75005 Paris, France; rim.bechaieb@gmail.com; 2 Department of Chemistry, College of Science, King Saud University, P.O. Box 22452, Riyadh 11495, Saudi Arabia; moraby@ksu.edu.sa; 3 Department of Pharmaceutical Chemistry, College of Pharmacy, King Saud University, P.O. Box 2457, Riyadh 11451, Saudi Arabia; halrabiah@ksu.edu.sa; 4 Computational and Quantum Chemistry Group, Instituto Federal de Educação, Ciência e Tecnologia do Ceará, Campus Camocim, Camocim 62400-000, Ceará, Brazil; bruno.poti@fisica.ufc.br; 5 Institute for Inorganic Chemistry and Materials Chemistry, Department of Chemistry and Biochemistry, Faculty of Mathematics and Natural Sciences, University of Cologne, Greinstrasse 6, D-50939 Köln, Germany; mniazi1@smail.uni-koeln.de

**Keywords:** 2,3,5-Triphenyl-2*H*-tetrazol-3-ium, crystal structure, hirshfeld surface analysis, DFT benchmarking, solid-state DFT calculations

## Abstract

2,3,5-triphenyl-2*H*-tetrazol-3-ium (TPT) chloride was studied through a combination of theoretical methods and experimental data, revealing structural and physical-chemical properties of the hydrate salt, [TPT]Cl·H_2_O. The previously reported crystal structure was confirmed, but our study at lower *T* (100 K vs. 220 K) showed different positions for the two H_2_O molecules in the unit cell around the chlorides. One of them (Cl1) is found surrounded by the tetrazole units, which we call the “dry pocket”, in contrast to the other, Cl2, which is involved in a hydrogen bonding cluster that consists of chloride and two water molecules, referred to as the “wet pocket”. Hirshfeld surface analyses showed predominant H⋯H interactions, followed by C⋯H interactions (including C–H⋯Cl/O interactions), and H⋯Cl contacts, which represent the C–H⋯Cl2 hydrogen bonds. Density functional theory (DFT) and (time-dependent) TD-DFT calculations on a molecular model of the compound, benchmarking the three functionals B3LYP, CAM-B3LYP, and PBE1PBE, found excellent agreement with experimental solution data when using the CAM-B3LYP function. UV-Vis absorptions observed at 320 nm, 245 nm, and 204 nm (in MeOH solution) were quite accurately reproduced and assigned. The observed bands were assigned to mixed HOMO–n⟶LUMO+m transitions, involving in all cases the LUMO+1 for the most intense band at 245 nm. Solid-state calculations on the GGA (PBE) level of theory using the CASTEP code and including the Tkatchenko–Scheffler (TS) scheme for the description of long-range interactions gave a good match for the calculated electronic band gap in the solid-state of 3.54 eV compared with the experimental value of 3.12 eV obtained through the Tauc plot method.

## 1. Introduction

**Scheme 1:**
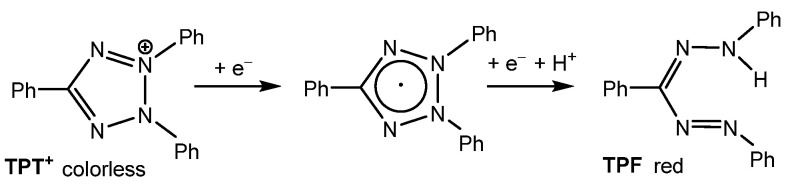
Reduction of 2,3,5-triphenyl-2*H*-tetrazolium [TPT]^+^ (**left**) to triphenylformazane (TPF, (**right**)).

2,3,5-Triphenyl-2*H*-tetrazol-3-ium [TPT]^+^ is a versatile cation that has been widely used for the structuring and stabilization of cationic species [1,2,3,4,5,6,7] and the detection of radiation damage [8,9,10]. However, the most frequent application of the commercially available chloride salt, [TPT]Cl (CAS No. 298-96-4), is its use as a staining agent in cell viability assays [11,12,13,14,15,16,17,18]. The staining process relies on the reduction of the colorless tetrazolium salt to the deep-red formazan (TPF) (Scheme 1) by mitochondrial dehydrogenases, which are key enzymes involved in the oxidation of organic compounds and cellular metabolism. This enzymatic reaction distinguishes metabolically active from inactive tissues. In necrotic regions, where these enzymes are either denatured or degraded, the tetrazolium salt remains unreacted [12,13,14,15,16].

This property has been particularly valuable in autopsy pathology, where [TPT]^+^ has been employed to detect myocardial infarctions. Viable heart muscle stains a deep red due to cardiac lactate dehydrogenase activity, whereas infarcted areas appear pale, reflecting the absence of enzymatic activity [17]. Very recently, [TPT]Cl was also used for the detection of electroporated areas in a potato model to study irreversible electroporation (IRE). Blackening or IRE-ablated areas is caused by an interplay of accumulated melanin and [TPT]^+^ [18].

Furthermore, [TPT]Cl has been widely employed as an analytical reagent in various potentiometric and spectrophotometric applications [19,20,21,22,23,24,25]. For instance, a liquid [TPT]^+^ ion-selective electrode (ISE) has been produced, utilizing the [TPT]^+^-phosphotungstate salt [TPT]_3_[P(W_3_O_10_)_4_] dissolved in 2-nitrotoluene. This electrode has been applied for direct potentiometric determination of [TPT]^+^ in aqueous solutions and for indirect potentiometric assays of ascorbic acid (vitamin C) in pharmaceutical preparations [19]. Similarly, potentiometric polyvinyl chloride (PVC) membrane sensors have been designed for the determination of [TPT]Cl and ascorbic acid. These sensors incorporate [TPT]^+^-tetraphenylborate ion pairs as ion exchange sites in a PVC matrix, with dioctylphthalate serving as a plasticizer. These devices have been used for indirect potentiometric determination of ascorbic acid in pharmaceutical formulations [20]. Another PVC membrane electrode, selective for pentachlorophenolate ions, has been constructed using [TPT]^+^-pentachlorophenolate ion pairs as the electroactive material. This sensor has demonstrated utility in the determination of pentachlorophenol in water, soil, and wood samples [21]. Additionally, a carbon paste sensor has been fabricated for potentiometric determination of sulfaquinoxaline. This sensor employs ion pairs generated from sulfaquinoxaline sodium and [TPT]Cl and has been successfully applied to the analysis of sulfaquinoxaline in pharmaceutical solutions, blood serum, urine, and milk samples [22]. The specific ability of [TPT]^+^ in forming ion pairs for analytical determinations was also employed for the spectrophotometric determination of Mo(IV) via liquid–liquid extraction, for which ion pairs of [TPT]^+^ and [MoO_2_(DNC)_2_]^2−^ (H_2_DNC = 3,5-dinitrocatechol) were described [23]. Similarly, [TPT]Cl has been employed in a spectrophotometric method for the determination of Co(II), utilizing a liquid–liquid extraction system containing 4-(2-thiazolylazo)resorcinol and [TPT]Cl [24]. Furthermore, a liquid–liquid extraction method has been developed for the determination of Ga(III), based on the formation of ion pairs between [TPT]^+^ and [Ga(PAR)_2_]^–^ (H_2_PAR = 4-(2-pyridylazo)resorcinol-triphenyl) in CHCl_3_ [25].

In contrast to these important applications, fundamental structural, electronic, and spectroscopic studies on [TPT]Cl using experimental and/or quantum chemical calculation approaches are limited [8,26,27,28,29,30,31]. Herein, we report on a comprehensive combined experimental/theoretical study on this compound. We crystallized the monohydrate [TPT]Cl·H_2_O and studied the structural features of this compound by single-crystal X-ray diffraction and Hirshfeld Surface Analysis. Based on the crystal structure, we developed a density functional theory (DFT)-calculated structural dimeric model (content of the unit cell) of [TPT]Cl·H_2_O, benchmarking the performance of the B3LYP, CAM-B3LYP, and PBE1PBE functionals in the gas phase and in MeOH solution (PCM). From these models, we calculated UV-Vis absorption spectra using time-dependent DFT, and fundamental electronic parameters, so-called descriptors, and compared them to experimental data (XRD, UV-Vis, and data from others). Furthermore, we compared the results of a solid-state modeling of the crystal structure and the electronic band structure on the GGA (PBE) level of theory using the CASTEP code and including the Tkatchenko–Scheffler (TS) scheme with the experimental structure and the electronic band gap energy (Tauc plot method).

## 2. Results and Discussion

### 2.1. Sample Preparation and Characterization

Colorless crystals were obtained by recrystallizing commercially available [TPT]Cl from an MeCN solution. The elemental analysis (see Materials Section 3) fits the formula [TPT]Cl·H_2_O, ESI-MS (Figure S1, Supplementary Materials) shows the [TPT]^+^ cation. The melting point, the UV-Vis absorption (see later), and the FT-IR spectra (Figure S2, Supplementary Materials) agree with those previously reported [8,10,26,27,28], thus confirming the entity and purity of the compound. Specifically, the FT-IR spectrum shows a broad unresolved band peaking at 3330 cm^−1^, representing the O–H vibration modes of the co-crystallized water molecules. A more pronounced band system with a maximum at 3040 cm^−1^ is assigned to aromatic C–H vibrations of the [TPT]^+^ cation [6,8]. The intense, sharp peaks in the range 1500 to 600 cm^−1^ also stem from the [TPT]^+^ cation and agree completely with previous reports [6,8,10,26,27,28].

### 2.2. Single-Crystal X-Ray Structure Determination

**Figure 1:**
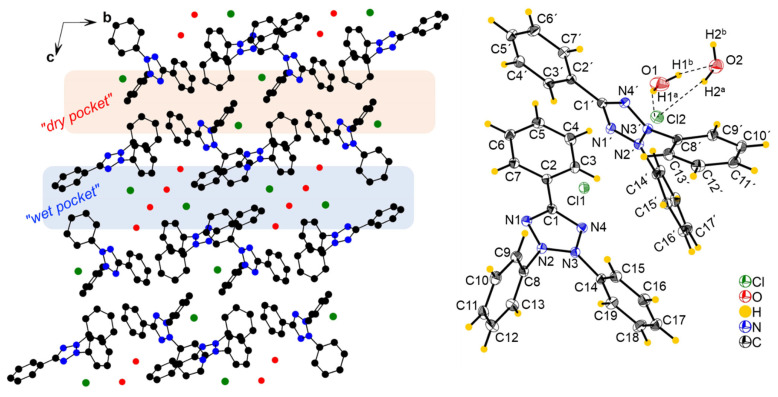
(**Left**): Crystal structure of [TPT]Cl·H_2_O viewed along the crystallographic *a* axis, with H atoms omitted for clarity. (**Right**): View of two [TPT]^+^ cations interacting with one of the Cl anions (Cl1), including the H atoms and hydrogen bonding. Ellipsoids are shown at a 50% probability level. Detailed hydrogen bonding information is provided in Table S2.

The compound [TPT]Cl·H_2_O crystallized in the triclinic space group *P*$\bar{1}$ with two crystallographically independent [TPT]^+^ cations, two Cl^−^ anions, and two H_2_O molecules in the unit cell (Figure 1, further figures in the Supplementary Materials, Figures S3 and S4, structure and refinement data in Table S1).

A previous structure determination of [TPT]Cl·H_2_O in 2005 gave the same space group (*P*$\bar{1}$), and the cell metrics are similar [31] (Table S1) and are thus isostructural. Specifically, both structure determinations reveal two distinct chloride anions. One of them is involved in a hydrogen-bonding cluster consisting of one chloride (Cl2) and two water molecules, while the other (Cl1) is not surrounded by water. We refer to these as the “wet pocket” and the “dry pocket”, respectively. Figure 1 shows that “wet pockets” extend over two Cl2^–^ chlorides and four water molecules and that the “wet” and the “dry pockets” separate alternating chains of [TPT]^+^ cations aligned along the crystallographic *b* axis. In keeping with the 2005 determination, the Cl^−^ in the “wet pocket” (Cl2) is found centered on the tetrazolium ring in a κ^5^-type interaction, while the Cl^−^ in the “dry pocket” (Cl1) is slightly offset from the center of the tetrazolium, bending towards N2 and N3 in an approximate κ^2^ + κ^3^ type of coordination (Figure 1). Additionally, the solvation of Cl2 results in decreased Cl⋯tetrazolium contacts [31]. We thus can confirm the essential bonding features of the 2005 structure determination, while the surroundings of the Cl1 anion differ markedly in that the N⋯Cl1 and H⋯Cl2 distances are much shorter, making the difference between the “wet” and the “dry” pocket larger than in the reported structure (Table 1). Furthermore, our analysis was conducted at 100 K, in contrast to the 2005 measurement at 220 K, and on different devices (Table 1). As a result, our data is generally more precise, while we do not claim our structure determination to be that of a new polymorph.

The structure of [TPT]Cl·2.5H_2_O, submitted in 2023 to the Cambridge Crystallographic Data Centre (CCDC 2314756) by Fronczek et al., differs markedly from ours, although the space group is the same (*P*$\bar{1}$) and the cell metrics are very similar (Table S1) [32]. The key difference is that this structure features only one type of chloride anion, which is involved in hydrogen bonding with the co-crystallized water. In other words, there is only one type of pocket, and this pocket is “wet”. Furthermore, the total amount of co-crystallized water accounts for 2.5 molecules. Consequently, the Cl⋯N distances are 3.944(1) Å and 3.813(1) Å, with the Cl^−^ residing in a position near the N2–N3 bond of the tetrazolium. This arrangement is very similar to the structure of [TPT]Br·EtOH (orthorhombic *P* 2_1_2_1_2_1_), in which the Br^−^ is engaged in EtO–H⋯Br hydrogen bonding and resides near the N2–N3 bond of the tetrazolium, with Br⋯N distances ranging from 3.472(2) Å to 3.564(2) Å [33].

For the corresponding [TPT]Cl·MeOH and [TPT]Cl·MeCN solvates, the Cl⋯tetrazolium interactions exhibit slight differences in orientation but are generally of the same type, with similar Cl⋯N distances as in the previously reported [TPT]Cl·H_2_O structure (at 220 K) [31] and those of our structure determination (at 100 K). Furthermore, the solvate-free 2,3,5-triphenyl-2*H*-tetrazol-3-ium iodide (*P*2_1_/c) shows two phenyl-tetrazolium moieties sandwiching the I- a bit similar to the dry pocket of Cl1 in our structure [34].

A previously reported structure optimization for the solvent-free (anhydrous) [TPT]Cl using density functional theory (DFT) methods at the B3PW91/6-311+G** level in the gas phase aligns qualitatively well with the experimentally observed Cl⋯tetrazolium interactions in the reported structure of the hydrate [TPT]Cl·H_2_O [31]. The optimization yielded an overall Cl⋯CN_4_ plane distance of 3.076 Å and a ring slippage beyond the N2–N3 bond, consistent with an unequivocal κ^2^-type interaction. This is remarkable, as this study and all previous reports have used the solvent-free [TPT]Cl for structure optimization [26,27,31]. Thus, none of them matches the real experimental structure determined by us and earlier in 2005 [31]. Especially, they fail to model the “wet pocket” of the structure (see later, DFT calculations).

Similar X^–^⋯^+^CN_4_ tetrazolium contacts with X = O, S, or Se were recently reported for TPP tetraphenyldichalcogenoimidodiphosphinates and identified as mainly electrostatic through DFT calculations, including non-covalent interaction (NCI) analysis [3].

### 2.3. Hirshfeld Surface Analysis and 2D Fingerprints

**Figure 2:**
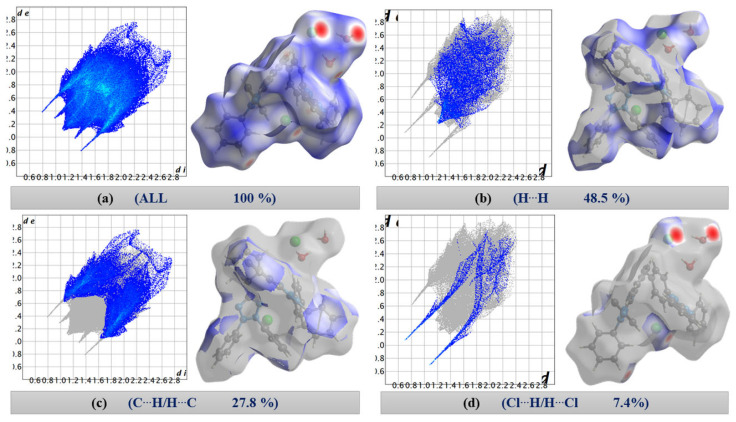
Two-dimensional fingerprint plots. (**a**) All contacts (100%), (**b**) H⋯H, (**c**) C⋯H/H⋯C, and (**d**) Cl⋯H/H⋯Cl contacts in [TPT]Cl·H_2_O.

The 2D fingerprint plots of [TPT]Cl·H_2_O (Figure 2) quantitatively illustrate the contributions of various interactions within the crystal structure, with surfaces mapped across a d_norm_ range from −0.4755 (red) to 1.4950 (blue) (Figure 2a).

On first view, the bright red areas surrounding the O and Cl atoms represent the presence of H⋯Cl and H⋯O hydrogen bonding interactions of the “wet pocket”. However, the most abundant contributions, with 48.5% and 27.8% (Figure 2), respectively, of the total Hirshfeld Surface Area (HSA), are the H⋯H and C⋯H interactions (Figure 2b,c). This is attributed to the high presence of H atoms on the molecular surface, as a result of the four Ph substituents dominating the surface of the [TPT]^+^ cation. The H⋯Cl contacts are depicted as a pair of pronounced spikes in the 2D fingerprint plot (Figure 2d), representing the C–H⋯Cl and O–H⋯Cl hydrogen bonding interactions. This interaction constitutes the third most significant contribution, accounting for approximately 7.4% of the total contacts. The H⋯N and H⋯O interactions, which correspond to O–H⋯N and O–H⋯O hydrogen bonding, represent the fourth and fifth most abundant contacts (Figure S5). Both H⋯Cl and H⋯O interactions characterize the “wet pocket” of the structure.

In addition to the d_norm_, the shape index and curvedness surfaces were created to study possible π-stacking in the structure. The shape index and curvedness values range from −1 to 1, and from −4 to 0.4, respectively (Figure S6). The green flat region in the curvedness and the blue and red triangles in the shape index help in identifying Cl···tetrazolium contacts (Figure S6).

### 2.4. DFT Calculations in the Gas Phase, Solution, and in the Solid—Benchmarking to Structure Data

DFT-calculated optimized geometries for the dimeric model (content of the unit cell; see Materials and Methods Section 3) of [TPT]Cl·H_2_O using the PBE1PBE, B3LYP, and CAM-B3LYP hybrid functionals in the gas phase, as well as in MeOH solution (PCM), using the CAM-B3LYP functional, were benchmarked against the experimental data from X-ray diffraction (Table 1 and Table S2). The choice of the functionals is motivated by benchmarking studies on related organic salts and ionic solids, which demonstrate B3LYP’s reliable structural and spectral predictions [35] and CAM-B3LYP’s accuracy for long-range excitations [36]. The PBE1PBE functional [37] was included in view of its applicability in solid-state systems. Similar DFT/TD-DFT studies on tetrazolium and formazan salts, for example, 3-substituted phenyl-3,5-diphenylformazans (PBE1PBE/6-311G(2d,2p), PCM) and diazonium “fast dye” salts (PBE0/6-311G(2d,2p), PCM), reproduced experimental UV-Vis absorption λ_max_ within 0.1 eV in MeOH [38].

**Figure 3:**
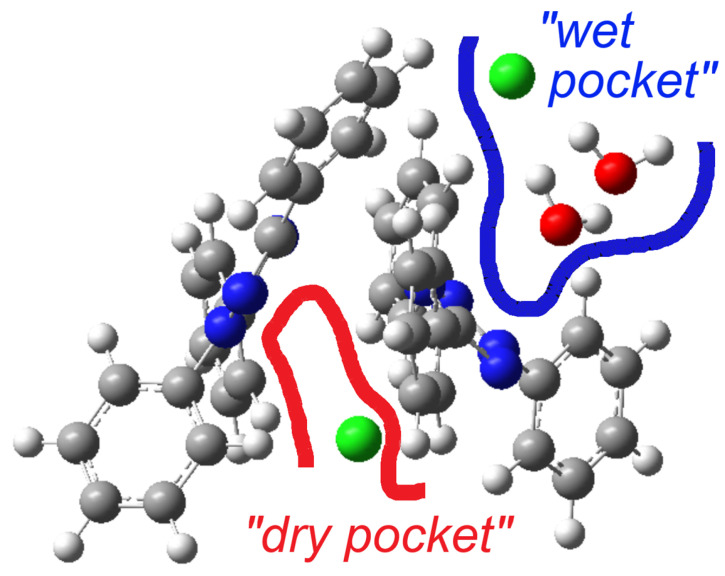
DFT-optimized geometry at the CAM-B3LYP level of the asymmetric unit of [TPT]Cl·H_2_O.

Starting from the unit cell contents with two [TPT]^+^ cations, two Cl^−^ anions, and two H_2_O molecules for the gas phase/solution calculations, all three functionals allowed modeling of essential features of the experimental structure, including the two different Cl^−^ ions and the “dry pocket” and “wet pocket” (Figure 3 and Figures S7–S9; selected data in Table 1, complete data in Table S3, coordinates in Tables S8–S13). While the intramolecular bonding parameters within the [TPT]^+^ core are very similar for the three different functionals, the intermolecular contacts, such as Cl⋯N and Cl⋯H, reveal marked variations between the datasets and also show quite poor agreement with the experimental data (Table S3), especially for the Cl1⋯N1 distance. Calculated root mean-square deviations (RMSD) (Figure S10) illustrate these deviations.

In contrast to this, using CAM-B3LYP allows better modeling of the “wet pocket”, which deems us important to model the electronic properties of the compound. Table 1 and Table S4 also show that the optimized geometry in MeOH shows superior agreement with the experimental data for both the “dry pocket” and “wet pocket,” with the exception of the O2⋯H1b interaction between the two water molecules, which can be attributed to solvation effects by MeOH. Working with only the asymmetric unit provides a reliable, although not perfect, description of the intrinsic molecular structure, and the resulting deviations from experimental values are consistent with the expected limitations of this approach.

To evaluate the basis-set sensitivity of the optimized geometries, we additionally optimized the dimer in the gas phase using the geometry-oriented pecG-2 basis set [39]. The method turned out to be far more time-consuming than 6-311+G(d), while the resulting structural parameters were not superior (Table S3, optimized Cartesian coordinates in Table S12). This stands in contrast to the recently reported superior performance of pecG-2 over 6-311+G(d) for geometry modeling of small organic molecules [39]. Probably, the ionic and dimeric character of our [TPT]Cl·H_2_O model makes a decisive difference to the molecules modeled in this work. Although pecG-2 is designed to improve gas-phase equilibrium bond lengths, our benchmarking against experiment shows that CAM-B3LYP/6-311+G(d) with implicit MeOH still provides the closest agreement.

As pointed out above, the recently reported geometry optimization of [TPT]Cl using B3LYP/6–311++G(d,p) functionals in the gas phase or with MeOH as solvent, came to very similar bonding parameters within the [TPT]^+^ core but failed to model the “wet pocket” [26]. This is also true for previous calculations on the anhydrous [TPT]Cl using the B3PW91/6-311+G** functional [31], and PM3 semi-empirical calculations [27].

The optimized lattice parameters from the solid-state calculations using the CASTEP code on GGA (PBE) [40] level of theory and including the Tkatchenko–Scheffler (TS) scheme of dispersion forces [41], showed very small deviations of 0.0024 Å for the crystallographic *a* axis, −0.0104 Å for *b*, and −0.0578 Å for *c* from the experimental data with a maximum error value of 0.34% (Table S3). Table 1 shows a very good agreement of the calculated data for the wet pocket with the experimental data.

### 2.5. Experimental and TD-DFT-Calculated UV-Vis Absorption Spectra in Solution

**Figure 4:**
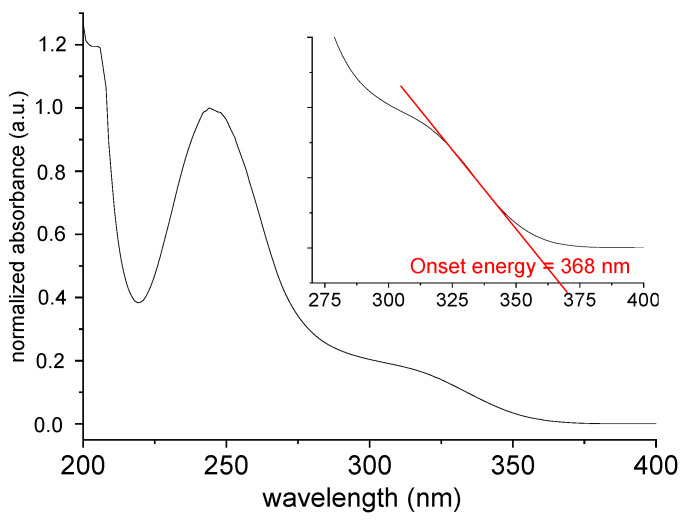
Normalized UV-Vis absorption spectrum of [TPT]Cl·H_2_O in MeOH.

The UV-Vis absorption spectrum of [TPT]Cl·H_2_O in MeOH solution shows two intense maxima at 204 and 245 nm alongside a broad shoulder at 320 nm (Figure 4) in keeping with previous reports [26,27,28,29]. These studies also showed that the absorption maxima depend slightly on the solvent polarity.

Based on the geometry optimizations described above (Section 2.4, we carried out time-dependent density functional theory (TD-DFT) to calculate UV-Vis absorption spectra of [TPT]Cl·H_2_O employing the B3LYP, CAM-B3LYP, or PBE1PBE functionals in the gas phase and the CAM-B3LYP functional in MeOH. The results are summarized in Table 2 (for figures, see Figures S11–S14). Figure S15 in the Supplementary Material shows a superposition of calculated spectra for different functionals to allow comparison of their performance. Qualitatively, all three functionals in the gas phase reproduced the experimental absorption spectrum (Table 2, Figures S11–S14). The energy of the central absorption peaking at 245 nm is best matched using the B3LYP functional. PBE1PBE lies only slightly higher for this band but matches the absorption at 204 nm pretty well. All three functionals located the long-wavelength maximum at around 300 nm. The relative intensity of the first two bands, as expressed by the oscillator strength, is best matched using B3LYP. Thus, in the gas phase, the results are too similar to allow a correlation of the “deviations from the experimental data” with the characteristics of the functionals (see above).

The three methods completely disagree on the involved transitions (Figures S11–S14). B3LYP calculations yielded the HOMO–18⟶LUMO transition as responsible for the long-wavelength absorption, while CAM-B3LYP defines this transition as HOMO–8⟶LUMO, and PBE1PBE as HOMO–6⟶LUMO+1. The three methods agree that the HOMO⟶LUMO transition has an oscillator strength close to zero and was excluded in our primary analysis. Thus, very probably, it does not play a role in the light absorption of [TPT]Cl·H_2_O. The most intense absorptions at 245 nm are due to transitions with very mixed character, except for PBE1PBE, in all cases involving the LUMO+1.

In MeOH, the PCM-solvated CAM-B3LYP calculation reproduces the experimental intensity pattern and partially corrects wavelength errors found in the gas phase. The long-wavelength band appears at 295 nm with an oscillator strength *f* = 0.12 and a modest 5 nm hypsochromic shift and a two-fold gain in intensity compared to the gas phase calculations. A detailed look at the involved orbitals shows that the dominating π⟶π* character of the gas phase calculated transitions (Figure S12) is now Cl1⟶π* charge transfer in MeOH (Figure S13), in keeping with the polar medium. A mixed π⟶π*/Cl⟶π* band is predicted at 240 nm (*f* = 0.29) and matches well to the experimental value of 245 nm. This time, also the “wet-pocket” Cl2 is involved. The 40% increase in oscillator strength reflects enhanced transition dipole coupling as solvent polarity reinforces the mixed character. A more pronounced bathochromic shift is observed for the high-energy manifold (230 nm, *f* = 0.20), indicating appreciable charge-transfer character in the mixed π⟶π*/Cl⟶π* transition, stabilized by MeOH. Despite these improvements, the shoulder calculated at 295 nm remains ≈30 nm blue-shifted from the experimental 325 nm feature, underscoring the known limitation of continuum models in strongly protic solvents. Explicit hydrogen bonding or polarizable embedding is often required for quantitative agreement. The full explicit-solvent treatment is reserved for future work focusing on quantitative solvatochromism.

Previously reported PM3-based Hartree-Fock calculations on [TPT]Cl showed the main absorption maximum at 200 nm, and weaker long-wavelength maxima at 253, 284, 320, and 356 nm, which do not fit well with the experimental spectrum [27]. Recently reported TD-DFT calculations on [TPT]Cl using the B3LYP/6–311++G(d,p) functional located the HOMO⟶LUMO transition at 410 nm and the most intense band at 261 nm with a mixed HOMO–1⟶LUMO+1 (87%), HOMO⟶LUMO+6 (10%) transition [26]. This is completely different from the results of our calculations using the same functional (Table 2). While we excluded the HOMO⟶LUMO transition from Table 2 for its virtually zero oscillator strength, we were able to match the main maximum at 245 nm very closely and assigned it to a mixed HOMO–6⟶LUMO+5/HOMO–4⟶LUMO+14 transition. The differences between our results and those of the previous reports are very probably due to our modeling of the hydrate [TPT]Cl·H_2_O, as all previous calculations were carried out on the anhydrous [TPT]Cl. Especially, the “wet pocket” of [TPT]Cl·H_2_O makes an enormous difference for the electrostatic potential of the Cl2^–^ anion and its interaction with the [TPT]^+^ cation. Very probably, our empirical dispersion corrections using the DFT-D3(BJ) method [42] helped further to match the experimental data.

The close agreement of the CAM-B3LYP/MeOH results obtained with the unified experimental geometry of the asymmetric unit with both the experimental results and our initial findings confirms the robustness of our computational strategy and validates the molecular orbital analysis.

In addition to the TD-DFT-calculated optical transitions, we also calculated the character and energies of the frontier orbitals and so-called global reactivity descriptors in the gas phase and solution [43,44,45,46]. The results are provided in the Supplementary Materials. In brief, when comparing the long-wavelength absorption energy of 3.87 eV (320 nm) with DFT-calculated gap energies, the CAM-B3LYP-calculated value of 4.29 eV in the gas phase and 3.97 eV in MeOH are in good agreement, whereas B3LYP and PBE1PBE severely underestimate the gap (1.39 and 1.85 eV, respectively) (Table S6). The HOMO is primarily localized on the Cl2 chloride anion (p orbital), with significant contributions from the two water molecules (Figure S16). This represents the nucleophilic part of the compound, but also confirms the important role of water in contributing to the overall bonding pattern and the crystal lattice energy. The LUMO is delocalized over the [TPT]^+^ cation centering in the tetrazolium core, which is the electrophilic part of the compound. The DFT-calculated electron affinity *A* (−*E*_LUMO_) (Table S7) is lowest for the CAM-B3LYP calculations, and the values of 2.03 eV (gas phase) and 2.97 eV (MeOH-PCM) are in line with the facile reduction of [TPT]^+^ (e.g., for the biochemical and potentiometric applications) and the reported −0.5 V (vs. SCE) for the first reduction of [TPT]Cl in dimethyl sulfoxide (DMSO) solution from a cyclic voltammetry study [30].

### 2.6. Solid-State Diffuse Reflection Spectrum and Calculations on the Electronic Structure

**Figure 5:**
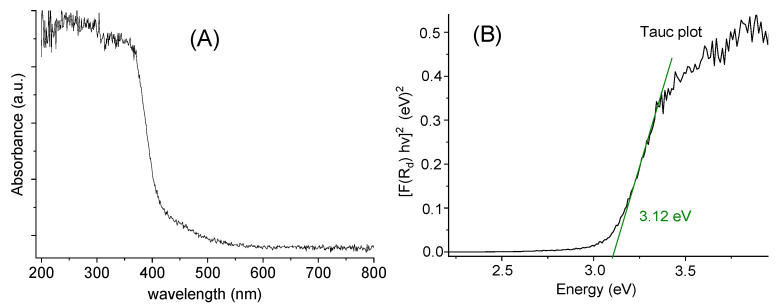
Diffuse reflectance spectrum (**A**) and Tauc plot (**B**) for [TPT]Cl·H_2_O in the solid.

The diffuse reflectance spectrum (Figure 5A) shows an absorption cut-off at about 375 nm, which is quite similar to the onset energy for the absorption spectrum in MeOH solution (Figure 4). The so-called Tauc plot method (Figure 5B) gave an optical band gap of 3.12 eV in the solid.

**Figure 6:**
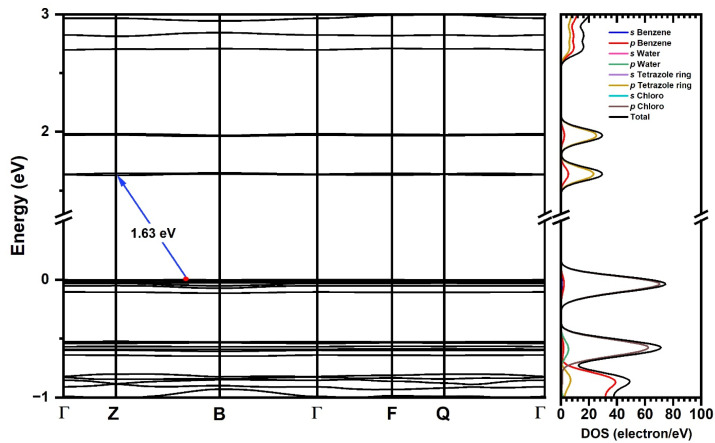
Kohn–Sham band structure and energy gap (*E*g) (blue arrow) for [TPT]Cl·H_2_O using the GGA+TS functional in the crystalline phase (**left**) and density of states of atomic/molecular fragments over energy (**right**).

The electronic structure was calculated using the same theory level that was applied for structural convergence. The Kohn–Sham (K-S) [47,48] band structure, which represents the electronic dispersion relation in terms of the k-points in the Brillouin zone, suggests an indirect gap, with the maximum value of the valence band (VBM) located between the Γ → B path, while the maximum of the conduction band (CBM) is located at the high symmetry point Z (Figure 6). The calculated band gap at the GGA+TS level of theory is 1.63 eV, differing from the experimental value of 3.12 eV by 1.49 eV.

To overcome this well-known K-S-intrinsic problem [49,50,51,52,53,54], we used the so-called Δ-sol method [54], with the fundamental gap calculated using Equation (1).

(1) $$E_{FG}=\left[E\left(N_{0}+n\right)+E\left(N_{0}-n\right)-2E\left(N_{0}\right)\right]/n$$

where *N*_0_ represents the number of valence electrons in the unit cell, *N* is a dependent functional parameter, and *n* is the charge in the unit cell corresponding to add/remove one electron and is defined as *n* = *N*_0_/*N* [54].

The thus calculated band gap energy is 3.54 eV, reducing the relative error from 1.49 eV (GGA+TS) to 0.42 eV. The same method was recently applied to calculate the band gap for the organic molecule dimethyl fumarate, increasing the classical Kohn–Sham-calculated 3.12 eV to 3.95 eV, which perfectly matches the experimental optical gap of 4.00 eV [55].

The electronic dispersion curves in Figure 6 reveal flat shapes of the frontier bands, indicating low mobility of the charge carriers in line with an insulating character of the material. The DOS calculations show that the valence band is dominated by the p orbitals of the Cl atoms, while for the conduction band, the p states of the tetrazole C and N atoms provide the major contributions. This suggests that electronic excitation occurs as charge transfer from a Cl p-type band to a C/N p-type band localized in the tetrazole, in keeping with our TD-DFT-calculated charge transfer transitions between p Cl and π* tetrazole orbitals (L’LCT) discussed in Section 2.4. Figure S16 shows the individual atom density of states.

**Figure 7:**
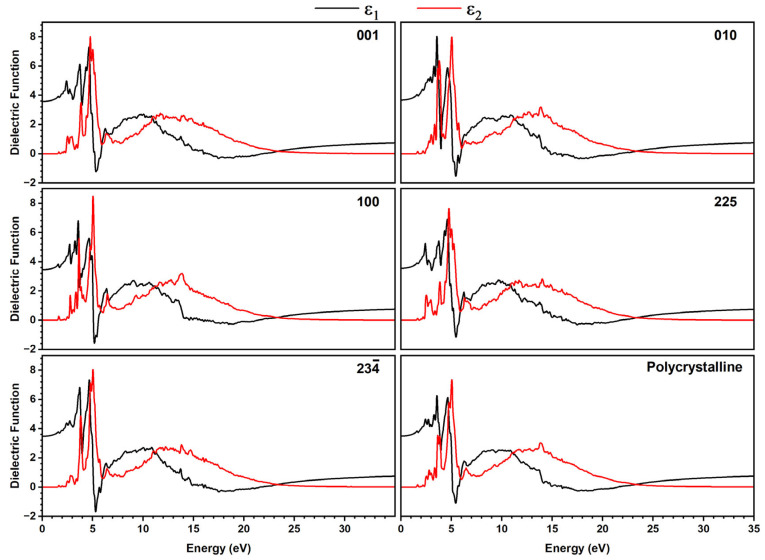
Dielectric function response plotted as real part ε_1_(ω) (black lines) and imaginary part ε_2_(ω) (red lines) in different directions of plane polarized light for [TPT]Cl·H_2_O using the GGA+TS functional in the crystalline phase. The directions 225 and 23$\bar{4}\;$ correspond to molecular planes defined by the tetrazole and phenyl rings.

The optical absorption profiles (Figure S17) and dielectric function, including the real part ε_1_(ω), which is related to radiation propagation and aligns with the energy storage ability of a material [53,56,57], and the complex (or imaginary) part ε_2_(ω), which is directly related to the absorption [58,59] (Figure 7), were calculated for different directions of plane-polarized light (001, 010, 100, 225, and 23$\bar{4}$), where the last two are referring to planes defined by the planar tetrazole and phenyl rings, respectively, and for polycrystalline material.

For both methods, we found sharp features at 2 to 7 eV and broad signals in the range of 8 to 25 eV for all directions of plane-polarized light, as well as for a polycrystalline sample. The only slight differences in the intensities of both signal groups, as well as the slightly different shapes of the signals for the different directions, point to only a small optical anisotropy.

A detailed view of the real part ε_1_(ω) showed that for ω = 0, in all directions, the maximum ε_1_ values are found in the range 3 to 5 eV. This region is dominated by π→π* transitions between Cl atoms and the aromatic tetrazole rings, in agreement with the DOS analysis. Moreover, all plots show positive ε_1_ values in the range 0 to almost 5 eV (~250 nm), suggesting propagation of electromagnetic waves with normal dispersion in this energy range, reaching from IR to the UV. In contrast, from 5 to 6 eV (~250 to 200 nm), the material reveals negative ε_1_ values pointing towards metal-type optical properties such as reflecting or absorbing propagating electromagnetic waves, with propagation direction represented by the Miller index in the crystallographic plane.

The recently reported DFT-calculated direction-dependent dielectric function responses for *L*-Ala·HCl are quite similar to those of [TPT]Cl·H_2_O with intense and sharp ε_2_ signals at around 6.5 eV, broad maxima between 7 and 14 eV, and a quite small anisotropy [56]. Comparison with non-salt derivatives such as *L*-Ala, *DL*-Ala, and β-Ala showed marked anisotropy for the latter, especially markedly varying peak intensities for the sharp low-energy components. Their GGA-TS-calculated indirect band gaps lie around 5 eV, with *L*-Ala·HCl showing the lowest with 4.68 eV [56]. A similar pronounced anisotropy as for the Ala derivatives was also found for the series of glycine polymorphs, including the hydrated glycine (Gly·2H_2_O) [60], *L*-histidine [53], and dimethyl fumarate [55].

Nevertheless, the electronic absorptions and the dielectric function responses for the above-named organic materials are very similar to that of [TPT]Cl·H_2_O, strongly supporting its insulating character, although the experimental optical band gap of 3.12 eV and the DFT-calculated (Δ-sol method) band gap of 3.54 eV might place the material into the vast group of wide-gap semiconductors [61].

## 3. Materials and Methods

### 3.1. Materials

#### 3.1.1. Commercially Available Materials

2,3,5-triphenyl-2*H*-tetrazol-3-ium chloride [TPT]Cl (≥98.0%, HPLC), KBr for IR pellets, MeCN, and MeOH as solvents were purchased from Merck (Darmstadt, Germany) and used without further purification.

#### 3.1.2. Sample Preparation of [TPT]Cl·H_2_O

Colorless crystals were obtained by recrystallization of 200 g (0.6 mmol) [TPT]Cl from a MeCN solution through slow evaporation of the solvent at room temperature. The resulting material was filtered off, rinsed with deionized water, and dried. Yield (collected crystals): 165 mg (0.46 mmol mol, 78%). Elemental analysis for [TPT]Cl·H_2_O (C_19_H_17_N_4_OCl, *M*_W_ = 352.81 g/mol): calculated (found) C: 64.62 (63.85), H: 4.81 (4.78), N: 15.87 (15.88)%. Melting point: 151 °C. ESI-MS(+) (*m*/*z*): found 299.38, calc. 299.13 for [TPT]^+^ (Figure S1). UV-Vis absorption (in MeOH, λ_max_ in nm): 204, 245, 320 sh. IR (KBr pellets, in cm^−1^): 3044 s, 2912 w, 1901 w, 1722 w, 1601 m, 1529 s, 1455 vs, 1362 m, 1297 m, 1163 vs, 1072 m, 998 s, 850 m, 770 vs, 723 w, 686 vs, 609 s, 505 m (Figure S2).

### 3.2. Experimental Methods

#### 3.2.1. General Instrumentation

The melting point was determined using a Gallenkamp apparatus (Gallenkamp & Co., London, UK). The FT-IR spectrum was recorded with a PerkinElmer FT-IR spectrometer (PerkinElmer, Shelton, CT, USA) using a KBr pellet. The mass spectrum was acquired using an Agilent Triple Quadrupole 6410 QQQ LC/MS system equipped with an electrospray ionization (ESI) source (Agilent, Santa Clara, CA, USA). Elemental analysis was performed with a PerkinElmer 2400 Series II CHNS/O analyzer. UV-Vis absorption spectra in MeOH solution at ambient temperature were recorded using a Shimadzu UV-1800 double-beam spectrophotometer (Shimadzu, Kyoto, Japan) with a quartz cell. Absorption and diffuse reflectance spectra on a solid sample were recorded using a Shimadzu UV 2600i UV-Vis spectrometer (Shimadzu, Kyoto, Japan).

#### 3.2.2. Single Crystal X-Ray Analysis

The crystal structure of [TPT]Cl·H_2_O was determined at 100(2) K using a Bruker D8 Venture diffractometer equipped with a Bruker Photon 100 CMOS detector and Mo Kα radiation (λ = 0.71073 Å) (Bruker, Rheinhausen, Germany). Data collection was performed using APEX3 v2015.5-2 software [62,63]. The structure was solved via dual-space methods using SHELXT [64] and refined with SHELXL 2017 employing full-matrix least-squares methods on *F*_0_^2^ ≥ 2σ(F_0_^2^) [65]. Non-hydrogen atoms were refined with anisotropic displacement parameters without constraints, while hydrogen atoms were incorporated using appropriate riding models. Graphics were generated using Diamond version 4.6.6 [66]. Crystallographic data for [TPT]Cl·H_2_O (CCDC 2373758) is available free of charge from the Cambridge Crystallographic Data Centre at https://summary.ccdc.cam.ac.uk/structure-summary-form or from the Cambridge Crystallographic Data Centre, 12 Union Road, Cambridge, CB2 1EZ UK (fax: +44-1223-336033 or e-mail: deposit@ccdc.cam.ac.uk).

#### 3.2.3. Hirshfeld Surface Analysis

Hirshfeld surfaces and their corresponding 2D fingerprint plots [67,68] were generated using CrystalExplorer 17 [68,69]. The structural input was provided in CIF format. The computation of normalized contact distances (d_norm_) incorporates the external distance (d_e_) measured from the Hirshfeld surface to the nearest nucleus outside the surface, the internal distance (d_i_) measured from the Hirshfeld surface to the closest nucleus within the surface, and the van der Waals radii [70,71]. 2D fingerprint maps, integrating d_e_ and d_i_ values, represent the intermolecular interactions [72,73].

### 3.3. DFT Calculations in the Gas Phase and in Solution

All computational analyses were performed using the Gaussian 16 software package [74]. Using the asymmetric unit of the [TPT]Cl·H_2_O crystal structure, which comprises two [TPT]^+^ cations, two Cl^−^ anions, and two water molecules (what we called “dimer”) in their experimentally determined positions, as the starting geometry for optimizations and frequency calculations, we benchmarked three different functionals. Initial calculations were performed in the gas phase using the widely utilized B3LYP functional [75,76], followed by the CAM-B3LYP functional [77,78] to account for long-range electronic effects. The PBE1PBE functional [37,79] was also included due to its applicability in solid-state systems. Initial calculations were conducted using the 6-311+G* basis set, with empirical dispersion corrections applied using the DFT-D3(BJ) method [42]. Later, we also applied the pecG-2 basis set [39]. Additionally, we carried out a geometry optimization in MeOH using the Polarizable Continuum Model (PCM) [80,81] at the CAM-B3LYP level.

Geometry optimizations for this molecular model of the compound [TPT]Cl·H_2_O were performed under the C_1_ symmetry group, ensuring that all structures converged to true minima on the potential energy surfaces, as confirmed by the absence of imaginary frequencies in the vibrational spectra. The optical properties and chemical reactivity in solution were modeled with full linear-response TD-DFT, which is implemented by default in Gaussian 16, including both excitation and de-excitation amplitudes, employing multiple exchange-correlation functionals (B3LYP, CAM-B3LYP, and PBE1PBE) on ground-state geometries optimized at the same level of theory in the gas phase and MeOH.

To explore the reactivity and stability, we and others employed a range of well-established descriptors, extensively discussed in previous studies [43,44,45,46]. These descriptors are calculated as follows [44,45]. Energy Gap: *E*g = *E*_LUMO_–*E*_HOMO_; Ionization Energy: *I* = −*E*_HOMO_; Electron Affinity: *A* = −*E*_LUMO_; Chemical Hardness: *η* = $\frac{\left(E_{LUMO}-E_{HOMO}\right)}{2}$; Chemical Potential: *µ* = $\frac{\left(E_{HOMO}+E_{LUMO}\right)}{2}$; Electrophilicity: *ω* = $\frac{{µ}^{2}}{2\eta }$; Electronegativity: *χ* = $\frac{\left(I+A\right)}{2}$; Softness: *S* = $\frac{1}{2\eta }$. HOMO = highest occupied molecular orbital, LUMO = lowest unoccupied molecular orbital.

### 3.4. Solid-State Calculations

Solid-state calculations were carried out using the CASTEP code [82,83], where both lattice parameters and atom positions were relaxed at the GGA (PBE) [40] level of theory. To improve the description of long-range interactions, the Tkatchenko–Scheffler (TS) [41] scheme of dispersion forces was applied in the calculations. Norm-conserving pseudopotentials [84] described the core electrons, adopting the valence configuration of 1s^1^ for H, 2s^2^2p^2^ for C, 2s^2^2p^3^ for N, 2s^2^2p^4^ for O, and 3s^2^3p^5^ for Cl atoms. The plane wave energy cutoffs adopted in the calculation were 830 eV, which have been demonstrated to be sufficient to reach the structural and electronic convergence for molecular crystals [53,54,55,56,85,86,87]. The Monkhorst–Pack [88] sampling of 2 × 1 × 1 was used to evaluate integrals in the reciprocal space. The unit cell was optimized for applying the convergence threshold of 0.5 × 10^−5^ eV/atom for total energy change, 0.01 eV/Å for total force per atom, 0.02 GPa for crystal pressure, and maximum atomic displacement smaller than 0.5 × 10^−3^ Å, with the Broyden–Fletcher–Goldfarb–Shannon (BFGS) [89] optimizer and self-consistent field convergence ensuring that the total energy per atom variation is smaller than 0.5 × 10^−5^ eV and the electron energy level changes smaller than 0.125 × 10 eV, within a convergence window of three cycles.

## 4. Conclusions

Herein, we report a comprehensive experimental/theoretical investigation on the structural and electronic properties of the 2,3,5-triphenyl-2*H*-tetrazol-3-ium chloride hydrate [TPT]Cl·H_2_O, the hydrate of [TPT]Cl, which is an important staining agent in cell viability assays and an important potentiometric sensor. The single-crystal X-ray diffraction analysis showed two distinct chloride environments. This includes Cl1 in a “dry pocket” showing an approximate κ^2^ + κ^3^ type interaction with tetrazolium N2 and N3 and closest Cl⋯N distances of 3.165(1) and 3.264(1) Å. At 3.366(1) and 3.412(1) Å, they are much longer for Cl2, which is residing in a “wet pocket” involved in hydrogen bonding with two H_2_O molecules and a tetrazolium κ^5^-type interaction. The Hirshfeld Surface Analysis underlined the huge differences between the two Cl^−^ anions. Experimental structural data and UV-Vis absorption data were used to benchmark the DFT and TD-DFT calculations in the gas phase and in solution (PCM) using B3LYP, CAM-B3LYP, and PBE1PBE functionals and the solid-state calculations on the GGA (PBE) level of theory using the CASTEP code and including the Tkatchenko–Scheffler (TS) scheme for the description of long-range interactions.

CAM-B3LYP in combination with the PCM model produces the best fit to experimental solution data. Specifically, the UV-Vis absorptions observed at 320 nm, 245 nm, and 204 nm in MeOH solution were reproduced at 300, 240, and 230 nm (MeOH, PCM model) and suggest a Cl⟶π* charge transfer character for the long-wavelength band at 300 nm and mixed π⟶π*/Cl⟶π* character for the strong absorptions at 240 and 230 nm. Remarkably, also the experimental molecular metrics from the crystal structure were reasonably matched, which is probably due to the chosen explicitly H_2_O-containing dimeric DFT model, in keeping with the unit cell contents.

The solid-state calculations on the GGA+TS level of theory even better matched the experimental structure data and gave a good match between the calculated and the experimental band gap energy, estimated through the Tauc plot method. Density of states, optical absorption spectra, and dielectric function response calculations strongly support the Cl-to-tetrazole charge transfer character of the UV-Vis absorption bands and confirm the insulating character of the material.

Thus, the combination of an appropriate molecular model and a suitable DFT method allowed us to closely model essential physicochemical properties of this compound, which define its broad application in many fields as outlined in the introduction.

## Figures and Tables

**Table 1:** Selected experimental and DFT-calculated equilibrium distances for [TPT]Cl·H_2_O ^a^.

Distances (Å)	Experimental	Calculated	Calculated
**method**	sc-XRD	CAM-B3LYP 6-311+G**	GGA (TS) ^c^
**medium**	MeOH ^b^	MeOH	Solid-state
Cl1⋯N1′	3.494(1)	3.447	3.637
Cl1⋯N2′	3.264(1)	3.281	3.387
Cl1⋯N3′	3.165(1)	3.267	3.281
Cl1⋯N4′	3.357(1)	3.402	3.501
Cl1⋯N1	3.549(1)	3.679	3.635
Cl1⋯N2	3.372(1)	3.350	3.434
Cl1⋯N3	3.429(1)	3.455	3.587
Cl2⋯C1	3.458(1)	3.483	3.589
Cl2⋯H1a	2.410(3)	2.460	2.332
Cl2⋯H2a	2.380(2)	2.234	2.292
O2⋯H1b	2.000(3)	1.873	1.860

^a^ The entries marked in gray represent the “wet pocket” of the structure. Experimental data from single-crystal X-ray diffraction. ^b^ Crystallized from MeOH. ^c^ Using the CASTEP code on GGA (PBE) level theory, including the Tkatchenko–Scheffler (TS) scheme. More data in Tables S2–S4.

**Table 2:** TD-DFT-calculated and experimental UV-Vis absorption data of [TPT]Cl·H_2_O ^a^.

		Calculated			Experimental
λ_max_ (nm)	Energy (eV)	Osc. Strength *f* ^b^	Coefficient	Transition	λ_max_ (nm) (rel. int.) ^c^
B3LYP (gas phase)					
301.88	4.11	0.0192 (0.15)	0.64256	HOMO-18⟶LUMO	320 (0.16)
244.00	5.08	0.1316 (1)	0.42220	HOMO-6⟶LUMO+5 HOMO-4⟶LUMO+14	245 (1)
221.82	5.59	0.0160 (0.12)	0.32191	HOMO-6⟶LUMO+9	204 (1.2)
CAM-B3LYP (gas phase)					
299.90	4.13	0.0514 (0.25)	0.51104	HOMO-8⟶LUMO	320 (0.16)
239.81	5.17	0.2015 (1)	0.32689 0.26157	HOMO-19⟶LUMO HOMO-8⟶LUMO+2	245 (1)
213.47	5.81	0.0656 (0.32)	0.35039 0.21722	HOMO-8⟶LUMO+1 HOMO-6⟶LUMO+7	207 (1.2)
CAM-B3LYP (MeOH)					
295.46	4.19	0.1205	0.51245	HOMO-4⟶LUMO	320 (0.16)
239.62	5.17	0.2865	0.28147 0.22766	HOMO-4⟶LUMO+1 HOMO-8⟶LUMO+3	245 (1)
229.81	5.39	0.2024	0.36581 0.13417	HOMO-8⟶LUMO+1 HOMO-4⟶LUMO+2	204 (1.2)
PBE1PBE (gas phase)					
300.69	4.12	0.0372 (0.28)	0.60609	HOMO-6⟶LUMO+1	320 (0.16)
247.90	5.00	0.1332 (1)	0.47803	HOMO-8⟶LUMO+2	245 (1)
206.92	5.99	0.0209 (0.16)	0.37581	HOMO⟶LUMO+19	204 (1.2)
CAM-B3LYP (MeOH) unified experimental geometry					
292.69	4.24	0.1382	0.59257	HOMO-3⟶LUMO	320 (0.16)
243.40	5.09	0.3131	0.43568	HOMO-17⟶LUMO+1	245 (1)
233.66	5.30	0.3465	0.29337 0.25541	HOMO-5⟶LUMO HOMO-6⟶LUMO	204 (1.2)

^a^ Using B3LYP, CAM-B3LYP, or PBE1PBE functionals. Absorption maxima λ_max_ in nm, oscillator strength, and major contributions. LUMO = lowest unoccupied molecular orbital; HOMO = highest occupied molecular orbital. Compiled are only the most probable transitions. ^b^ Relative intensities normalized to 1 for the band at around 240 nm in parentheses. ^c^ Experimental UV-Vis absorption maxima, spectrum recorded in MeOH solution (Figure 4); relative intensities in parentheses, normalized to 1 for the band at 245 nm.

## Data Availability

Data that is not included in the Supplementary Materials will be made available on request.

## Supplementary Materials

The following supporting information can be downloaded at https://www.mdpi.com/article/10.3390/molecules31010138/s1. Figure S1: ESI-MS of [TPT]Cl·H_2_O. Figure S2: FT-IR spectrum of [TPT]Cl·H_2_O. Figure S3: Crystal structure of [TPT]Cl·H_2_O viewed along the crystallographic *a* axis, including the hydrogen bonding network and hydrogen bond length (Å). Figure S4: Crystal structure of [TPT]Cl·H_2_O viewed along the crystallographic *b* axis, including the hydrogen bonding network. Figure S5: Total relative contribution of intermolecular contacts in [TPT]Cl·H_2_O to the Hirshfeld surface in %. Figure S6: Hirshfeld surfaces plotted over d_norm_, shape index, and curvedness for [TPT]Cl·H_2_O. Figure S7: DFT-optimized geometry of [TPT]Cl·H_2_O using the B3LYP functional. Figure S8: DFT-optimized geometry of [TPT]Cl·H_2_O using the CAM-B3LYP functional. Figure S9: DFT-optimized geometry of [TPT]Cl·H_2_O using the PBE1PBE functional. Figure S10: Root-mean-square deviation (RMSD) calculation between experimental (single-crystal XRD) and DFT-calculated equilibrium distances. Figure S11: DFT-calculated molecular orbitals and associated TD-DFT-calculated electronic transitions for [TPT]Cl·H_2_O using the B3LYP functional. Figure S12: DFT-calculated molecular orbitals and associated TD-DFT-calculated electronic transitions for [TPT]Cl·H_2_O using CAM-B3LYP functional. Figure S13: DFT-calculated molecular orbitals and associated TD-DFT-calculated electronic transitions for [TPT]Cl·H_2_O using the CAM-B3LYP functional in MeOH (PCM). Figure S14: DFT-calculated molecular orbitals and associated TD-DFT-calculated electronic transitions for [TPT]Cl·H_2_O using the PBE1PBE functional. Figure S15: TD-DFT-calculated UV-Vis absorption spectra on B3LYP, CAM-B3LYP, and PBE0 levels of theory in the gas phase and CAM-B3LYP in MeOH (PCM model). Figure S16: DFT-calculated frontier molecular orbitals and energy gap (*E*g) for [TPT]Cl·H_2_O using the CAM-B3LYP functional in the gas phase and in MeOH using the PCM method. Figure S17: Partial density of states (PDOS) for atoms in [TPT]Cl_._H_2_O using the GGA+TS functional in the crystalline phase. Figure S18: Absorption profiles in different directions of plane polarized light for [TPT]Cl_._H_2_O using the GGA+TS functional in the crystalline phase. Table S1: Selected crystallographic data of [TZT]Cl·H_2_O and derivatives. Table S2: Selected X–H⋯X and Cl⋯π distances (Å) of [TZT]Cl·H_2_O. Table S3: Selected experimental and DFT-calculated equilibrium distances for [TPT]Cl·H_2_O. Table S4: Selected experimental and calculated distances (Å) and angles (°) of [TZT]Cl·H_2_O using CAM-B3LYP (MeOH). Table S5: Experimental and DFT-optimized unit cell parameters, volume, and density of [TPT]Cl·H_2_O in the solid-state. Table S6: DFT-calculated HOMO and LUMO energies for [TPT]Cl·H_2_O and [TPT]Cl. Table S7: DFT-calculated global reactivity descriptors for [TPT]Cl·H_2_O and [TPT]Cl. Table S8: XYZ coordinates of the DFT-optimized structure of [TPT]Cl·H_2_O using B3LYP. Table S9: XYZ coordinates of the DFT-optimized structure of [TPT]Cl·H_2_O using PBE1PBE. Table S10: XYZ coordinates of the DFT-optimized structure of [TPT]Cl·H_2_O using CAM-B3LYP in the gas phase. Table S11: XYZ coordinates of the DFT-optimized structure of [TPT]Cl·H_2_O using CAM-B3LYP in MeOH. Table S12: XYZ coordinates of the DFT-optimized structure of [TPT]Cl·H_2_O using the CAM-B3LYP functional and pecG-2 basis set in the gas phase. Table S13: XYZ coordinates of the DFT-optimized structure of [TPT]Cl·H_2_O in the solid-state using GGA+TS.
