# Supplementary material for: What a Difference a Water Molecule Makes—A Combined Experimental/Theoretical Study on 2,3,5-triphenyl-2H-tetrazol-3-ium Chloride Hydrate in Solution and the Solid-State
Source: Molecules. 2025 Dec 31;31(1):138. doi: 10.3390/molecules31010138 (PMC12787995; doi:10.3390/molecules31010138)
Supplement: Supplementary file 1 [file molecules-31-00138-s001.zip › molecules-3967243-supplementary.pdf]

Supplementary Materials

for

What a difference a water molecule makes – A combined experimental/theoretical study on 2,3,5-triphenyl-2*H*-tetrazol-3-ium chloride hydrate in solution and in the solid-state

Rim Bechaieb[1], Maha F. El-Tohamy[2], Haitham AlRabiah[3], Gamal A. E. Mostafa[*,3], Bruno Poti e Silva [4], Maryam Niazi[4], Axel Klein[*,4]

[1] Laboratoire de Chimie Théorique, Sorbonne Université UPMC, Univ. Paris 06, UMR 7616, Paris F-75005, France, E-mail: rim.bechaieb@gmail.com, ORCID: 0000-0003-0500-8653 (R.B.)

[2] Department of Chemistry, College of Science, King Saud University, P.O. Box 22452, Riyadh 11495, Saudi Arabia, E-mail: moraby@ksu.edu.sa, ORCID: 0000-0001-9793-7761 (M.F.E.T.)

[3] Department of Pharmaceutical Chemistry, College of Pharmacy, King Saud University, P.O. Box 2457 Riyadh 11451, Saudi Arabia. E-mail: halrabiah@ksu.edu.sa (H.A.R.)

[4] Computational and Quantum Chemistry Group, Instituto Federal de Educação, Ciência e Tecnologia do Ceará, Campus Camocim, 62400-000, Camocim, Ceará, Brazil. Email: Bruno.poti@fisica.ufc.br, bruno.poti@ifce.edu.br, ORCID: 0000-0001-5830-8903 (B.P.S.)

[5] University of Cologne, Faculty for Mathematics and Natural Sciences, Department of Chemistry and Biochemistry, Institute for Inorganic and Materials Chemistry, Greinstrasse 6, D-50939 Köln, Germany. Email: mniazi1@smail.uni-koeln.de, ORCID: 0009-0006-3486-3690 (M.N.)

Contents:

Supplementary Results

1. DFT-Calculated Frontier Orbital Character and Energies in the Gas Phase and Solution

2. DFT-Calculated Global Reactivity Descriptors

Supplementary Figures

Figure S1. ESI-MS of [TPT]Cl·H_2O.

Figure S2. FT-IR spectrum of [TPT]Cl·H_2O.

Figure S3. Crystal structure of [TPT]Cl·H_2O viewed along the crystallographic *a* axis, including the hydrogen bonding network and hydrogen bond length (Å).

Figure S4. Crystal structure of [TPT]Cl·H_2O viewed along the crystallographic *b* axis, including the hydrogen bonding network.

Figure S5. Total relative contribution of intermolecular contacts in [TPT]Cl·H_2O to the Hirshfeld surface in %.

Figure S6. Hirshfeld surfaces plotted over d_{norm}, shape index, and curvedness for [TPT]Cl·H_2O.

Figure S7. DFT-optimized geometry of [TPT]Cl·H_2O using the B3LYP functional.

Figure S8. DFT-optimized geometry of [TPT]Cl·H_2O using the CAM-B3LYP functional.

Figure S9. DFT-optimized geometry of [TPT]Cl·H_2O using the PBE1PBE functional.

Figure S10. Root-mean-square deviation (RMSD) calculation between experimental (single-crystal XRD) and DFT-calculated equilibrium distances.

Figure S11. DFT-calculated molecular orbitals and associated TD-DFT-calculated electronic transitions for [TPT]Cl·H_2O using the B3LYP functional in the gas phase.

Figure S12. DFT-calculated molecular orbitals and associated TD-DFT-calculated electronic transitions for [TPT]Cl·H_2O using CAM-B3LYP functional in the gas phase.

Figure S13. DFT-calculated molecular orbitals and associated TD-DFT-calculated electronic transitions for [TPT]Cl·H_2O using the CAM-B3LYP functional in MeOH (PCM).

Figure S14. DFT-calculated molecular orbitals and associated TD-DFT-calculated electronic transitions for [TPT]Cl·H_2O using PBE1PBE functional in the gas phase.

Figure S15. TD-DFT-calculated UV-vis absorption spectra on B3LYP, CAM-B3LYP, and PBE0 level of theory in the gas phase and CAM-B3LYP in MeOH (PCM model).

Figure S16. DFT-calculated frontier molecular orbitals and energy gap (*E*g) for [TPT]Cl·H_2O using the CAM-B3LYP functional in the gas phase and in MeOH using the PCM method.
Figure S17. Partial density of states (PDOS) for atoms in [TPT]Cl·H_2O using the GGA+TS functional in the crystalline phase.
Figure S18. Absorption profiles in different directions of plane polarized light for [TPT]Cl·H_2O using the GGA+TS functional in the crystalline phase.

Supplementary Tables

Table S1. Selected crystallographic data of [TZT]Cl·H_2O and derivatives.
Table S2. Selected X–H···X and Cl···π distances (Å) of [TZT]Cl·H_2O.
Table S3. Selected experimental and DFT-calculated equilibrium distances for [TPT]Cl·H_2O.
Table S4. Selected experimental and calculated distances (Å) and angles (°) of [TZT]Cl·H_2O using CAM-B3LYP (MeOH).
Table S5. Experimental and DFT-optimized unit cell parameters, volume, and density of [TPT]Cl·H_2O in the solid state.
Table S6. DFT-calculated HOMO and LUMO energies for [TPT]Cl·H_2O and [TPT]Cl.
Table S7. DFT-calculated global reactivity descriptors for [TPT]Cl·H_2O and [TPT]Cl.
Table S8. XYZ coordinates of the DFT-optimized structure of [TPT]Cl·H_2O using B3LYP.
Table S9. XYZ coordinates of the DFT-optimized structure of [TPT]Cl·H_2O using PBE1PBE.
Table S10. XYZ coordinates of the DFT-optimized structure of [TPT]Cl·H_2O using CAM-B3LYP in the gas phase.
Table S11. XYZ coordinates of the DFT-optimized structure of [TPT]Cl·H_2O using CAM-B3LYP in MeOH.
Table S12. XYZ coordinates of the DFT-optimized structure of [TPT]Cl·H_2O using the CAM-B3LYP functional and pecG-2 basis set in the gas phase.
Table S13. XYZ coordinates of the DFT-optimized structure of [TPT]Cl·H_2O in the solid-state using GGA+TS.
References

Supplementary Results

1. DFT-Calculated Frontier Orbital Character and Energies in the Gas Phase and Solution

The experimental absorption spectrum shows a shoulder at 320 nm that translates to 3.87 eV and an extrapolation of the long-wavelength band to zero absorption gives an onset energy of 368 nm = 3.37 eV. The CAM-B3LYP-calculated gap energy *Eg* of 4.29 eV in the gas phase and 3.97 eV in MeOH are in good agreement with these values, whereas B3LYP and PBE1PBE severely underestimate the gap (1.39 and 1.85 eV, respectively) (Table S3).

On the other hand, from our TD-DFT calculated electronic transitions we excluded the HOMO→LUMO transition for its almost zero oscillator strength and the E_g of 4.29 eV translates into 289 nm (Figure S15), while the compound absorbs down to 368 nm. However, the overall numbers for the E_{HOMO} and E_{LUMO} from the CAM-B3LYP calculation are more convincing for the colorless and presumably insulating and material [TPT]Cl·H_2O than those using B3LYP or PBE1PBE.

The underlying orbitals of the CAM-B3LYP approach (Figure S15) show the HOMO located essentially on the Cl2 chloride anion (p orbital) with remarkable contributions from the two water molecules, underlining the importance of calculating [TPT]Cl·H_2O and not the anhydrous version [TPT]Cl. The LUMO is delocalized over the $[TPT]^+$ cation centering in the tetrazolium core. Although these DFT calculations must not be confused with the TD-DFT-calculated electronic transitions, we can state, that these two very different orbitals are in line with the findings by us and others [1] that the HOMO→LUMO transition is far too weak to be observable. The reported APFD/6–311++G(2d,p)-based calculations on [TPT]Cl gave a similar gap energy of 4.37 eV but localized both HOMO and LUMO on the $[TPT]^+$ core. Consequently, the HOMO→LUMO transition at 410 nm (3.02 eV) has a substantial oscillator strength and is visible in the calculated spectrum [1]. The semi-empirical PM3-based Hartree-Fock calculations for [TPT]Cl gave *Eg* values of about 9 eV and also localized both HOMO and LUMO on the $[TPT]^+$ core [2]. This value does not match at all with the experimental UV-vis absorption spectrum. However, it must be said that in this approach the calculated HOMO and LUMO energies and character and the calculated UV-vis absorption spectrum are methodically completely different to our DFT approach.

2. DFT-Calculated Global Reactivity Descriptors

To explore the reactivity and stability of the compound in solution, we employed a range of well-established descriptors (Table S4), extensively discussed in previous studies [3–6], including some reported on [TPT]Cl derivatives [1,2]. For the chemical potential μ of our dimeric model, all three methods gave similar values. The negative value of −4.18 eV in the gas phase and –4.96 eV in MeOH (CAM-B3LYP), indicates thermodynamical stability towards electrophilic or nucleophilic attack and thus general chemical stability. The value is in line with the HOMO–LUMO gap of 4.29 eV (gas phase) and 3.97 eV (MeOH-PCM) (Table 3). The electron affinity A ($-E_{LUMO}$) is lowest for the CAM-B3LYP calculations, and the values of 2.03 eV (gas phase) and 2.97 eV (MeOH-PCM) agree with the facile reduction of $[TPT]^+$ being a requisite for the biochemical and potentiometric applications and the reported –0.5 V (vs. SCE) for the first reduction of [TPT]Cl in dimethyl sulfoxide (DMSO) solution from a cyclic voltammetry study [7]. The recently reported APFD/6–311++G(2d,p)-based calculations on [TPT]Cl in the gas phase [1] fail to give reasonable parameters in the gas phase (I = 10.13 eV; A = 6.22 eV), while in MeOH, the values lie in a more realistic range (I = 7.59 eV; A = 3.26 eV) and also get closer to our results. However, –3.26 eV for the LUMO energy is pretty high in view of the facile chemical and electrochemical reduction. Following the CAM-B3LYP approach in MeOH, the derived parameters chemical hardness (η = 1.99 eV), electrophilicity (ω = 6.19 eV), and softness (S = 0.25 eV^{-1}) provide a realistic depiction of the chemical behavior of the compound. The softness value of 0.25 eV^{-1}, which is the reciprocal of the chemical hardness (1.99 eV), is in line with marked charge redistribution during interactions and high polarizability and fits very well to the easy electrochemical reduction. The electrophilicity of 6.19 eV agrees with the chemical potential μ and confirms the stability towards nucleophilic attack.

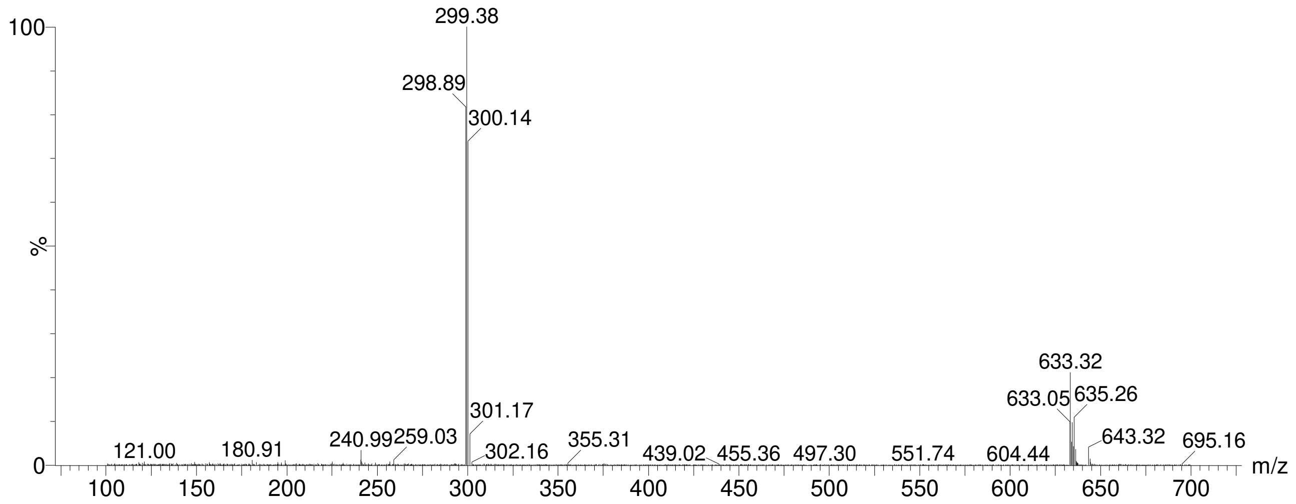

Figure S1. ESI-MS(+) of [TPT]Cl·H_2O.

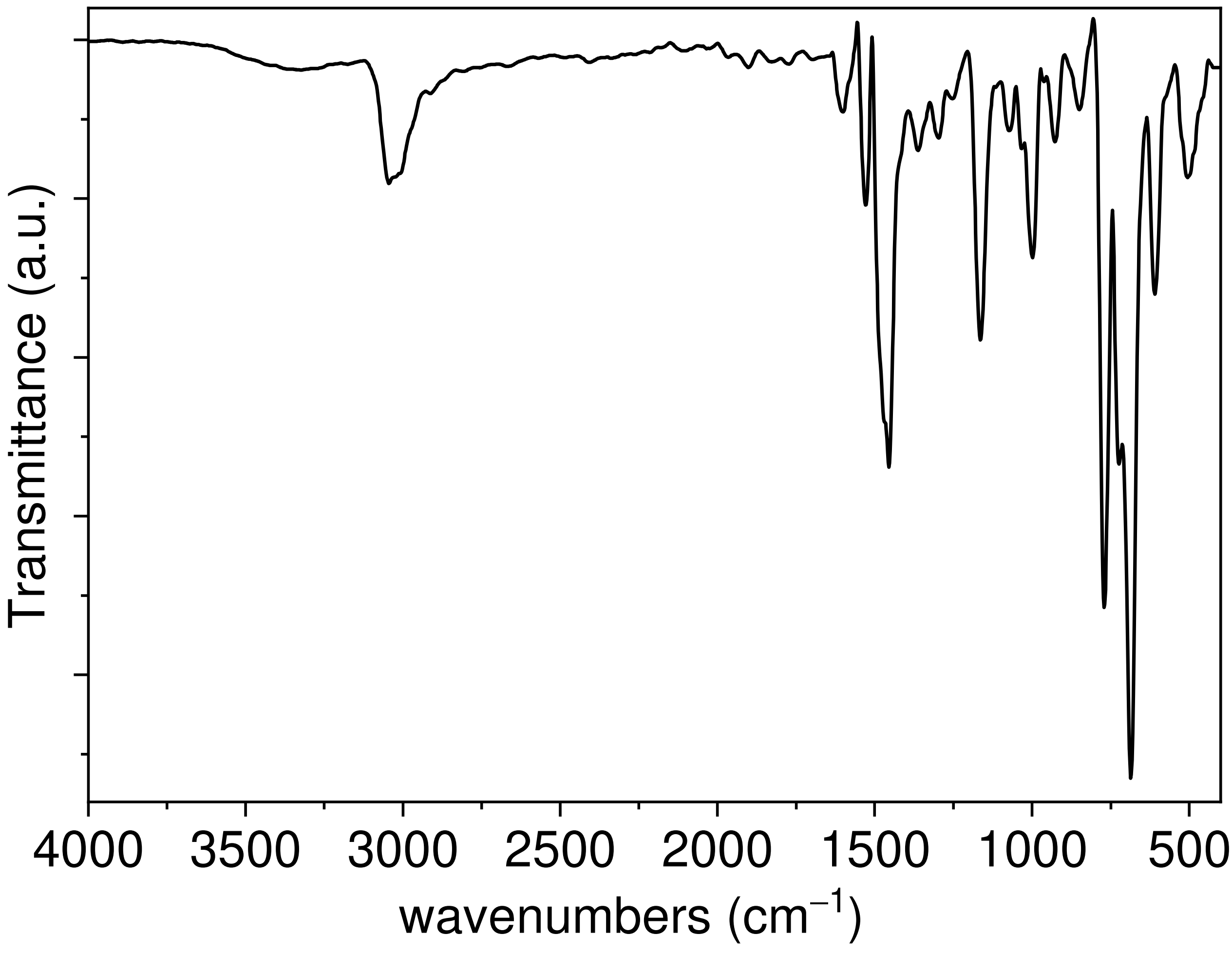

Figure S2. FT-IR spectrum of a sample of [TPT]Cl·H_2O as KBr pellet.

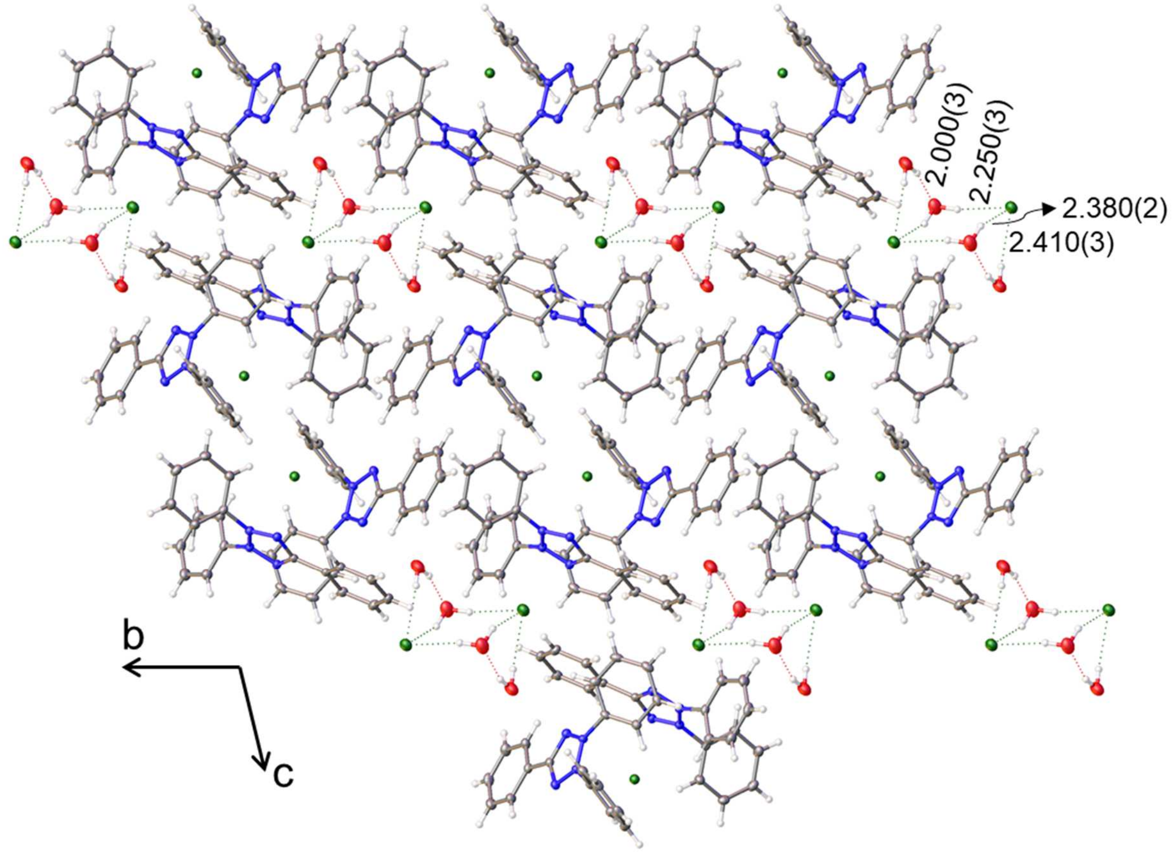

Figure S3. Crystal structure of [TPT]Cl·H_2O viewed along the crystallographic *a* axis, including the hydrogen bonding network with hydrogen bond length (Å).

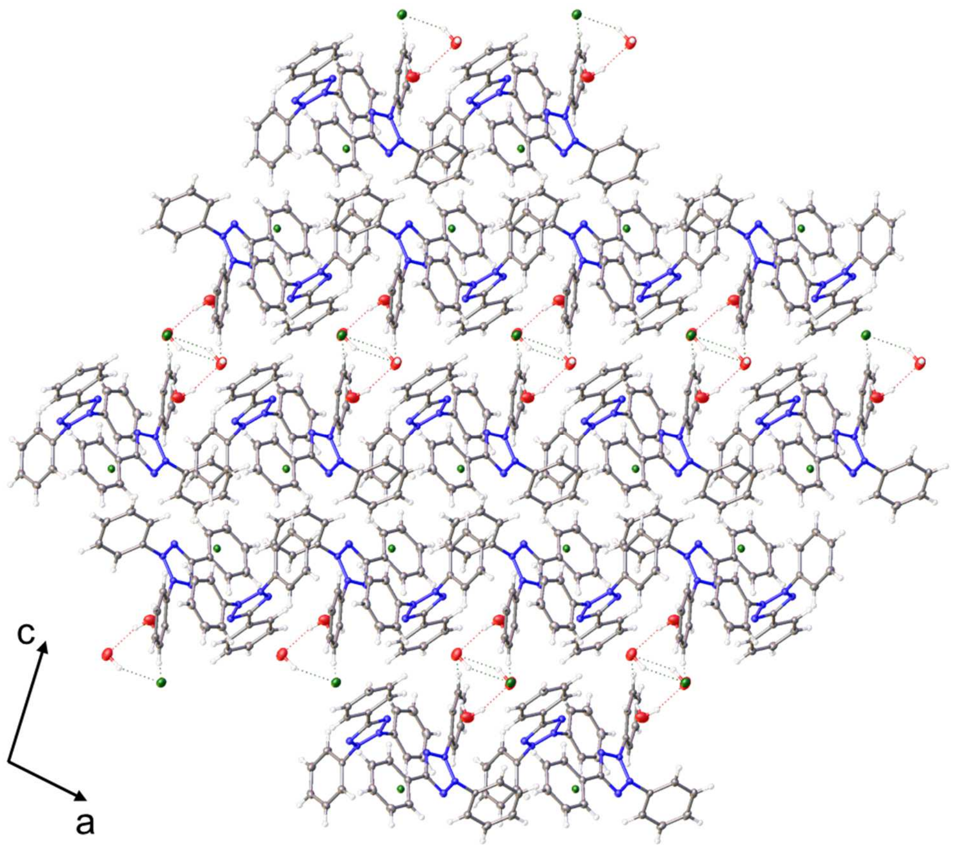

Figure S4. Crystal structure of [TPT]Cl·H_2O viewed along the crystallographic *b* axis, including the hydrogen bonding network.

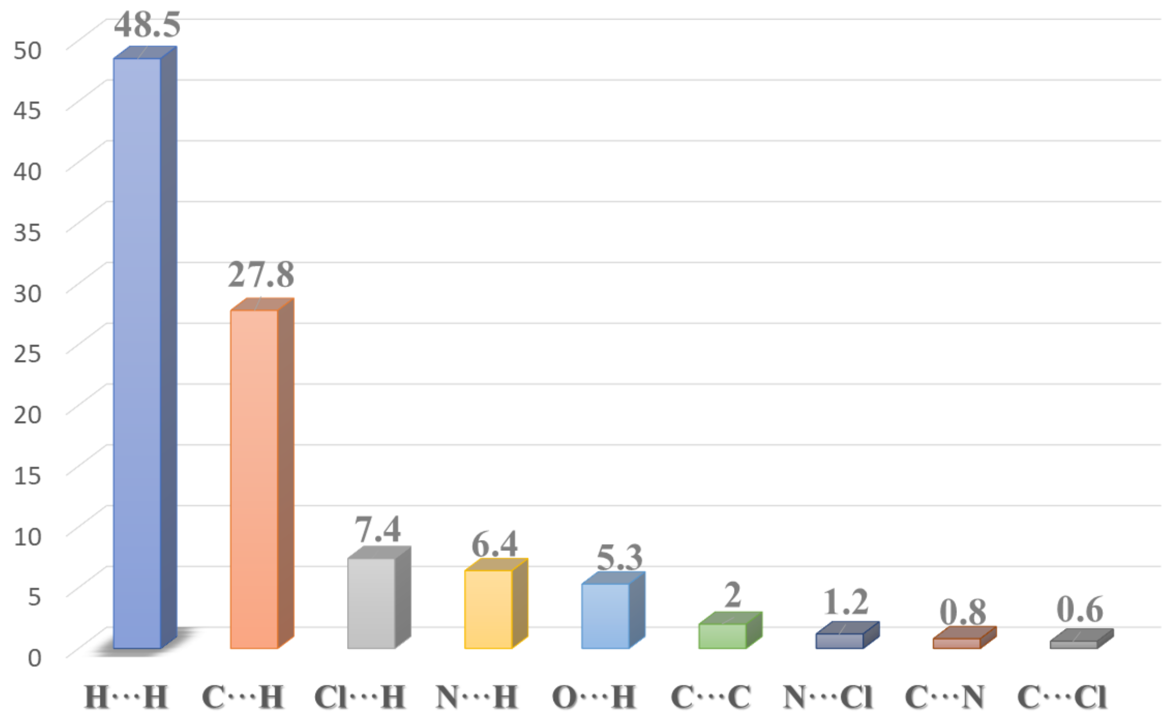

Figure S5. Total relative contribution of intermolecular contacts in [TPT]Cl·H_2O to the Hirshfeld surface in %.

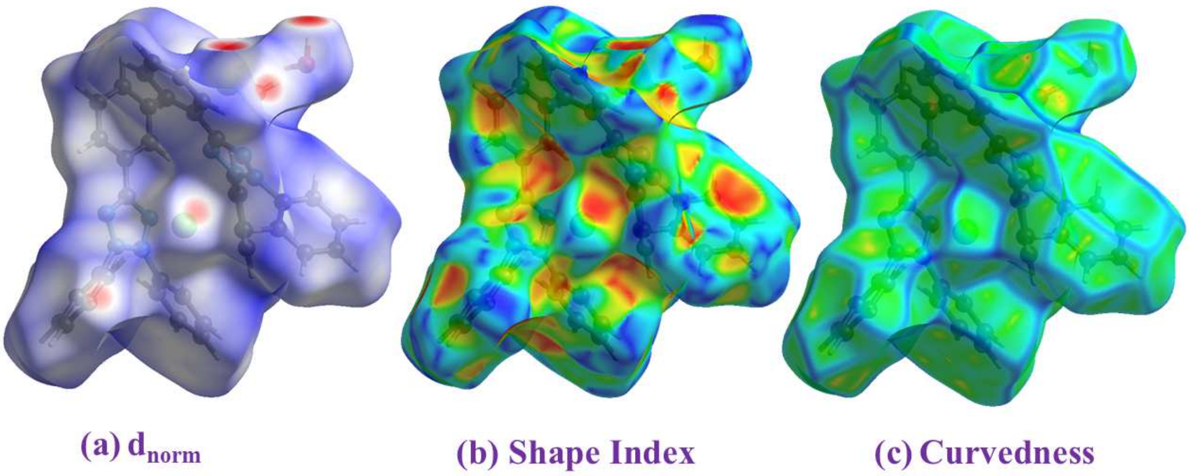

Figure S6. Hirshfeld surfaces plotted over d_{norm}, shape index, and curvedness for [TPT]Cl·H_2O.

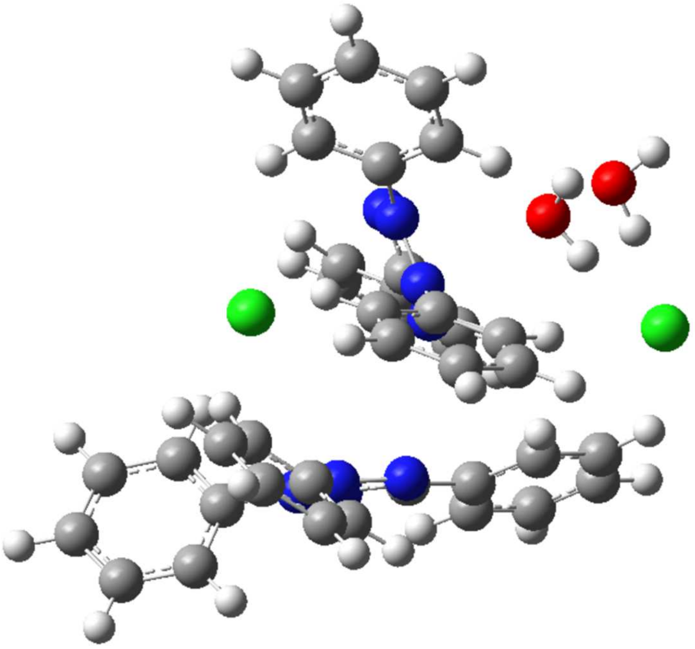

Figure S7. DFT-optimized geometry of [TPT]Cl·H_2O using the B3LYP functional in the gas phase.

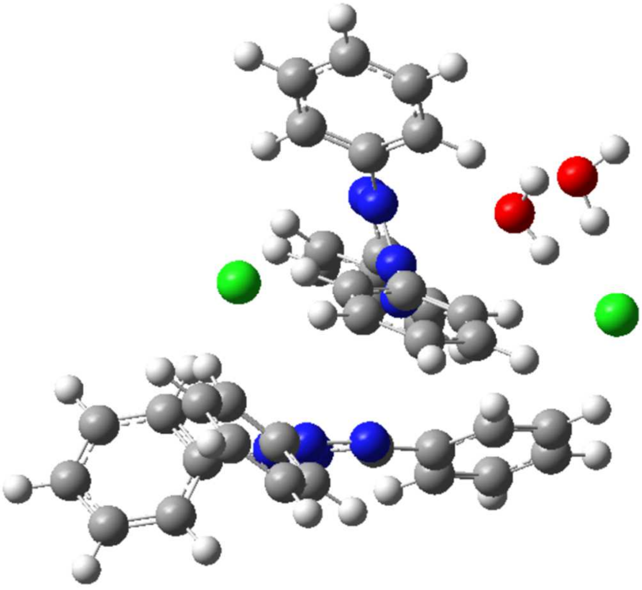

Figure S8. DFT-optimized geometry of [TPT]Cl·H_2O using the CAM-B3LYP functional in the gas phase.

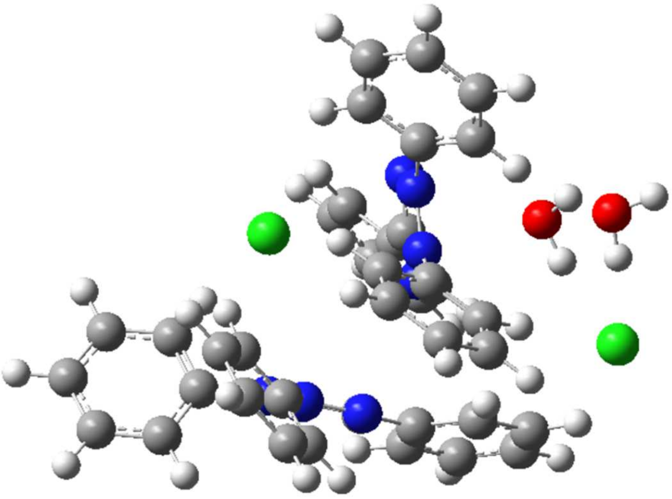

Figure S9. DFT-optimized geometry of [TPT]Cl·H_2O using the PBE1PBE functional in the gas phase.

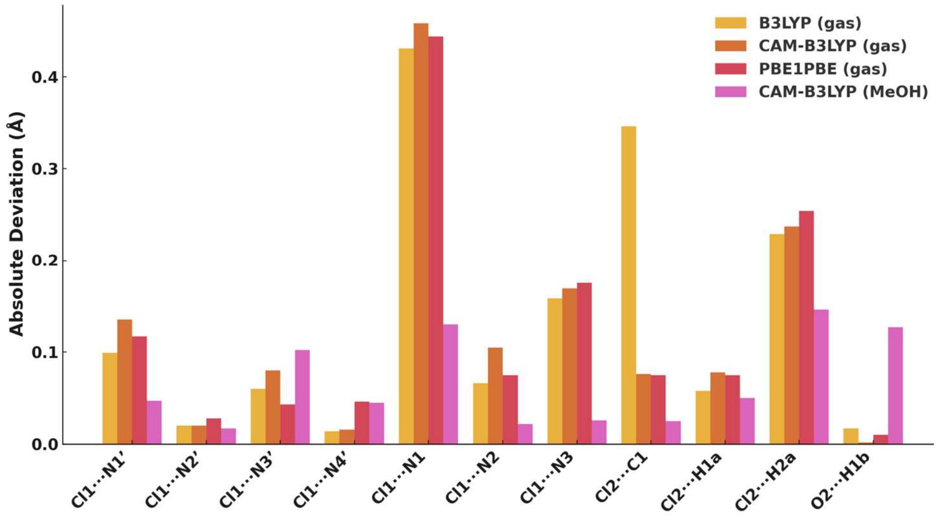

Figure S10. Root-mean-square deviation (RMSD) calculation between experimental (single-crystal XRD) and DFT-calculated equilibrium distances.

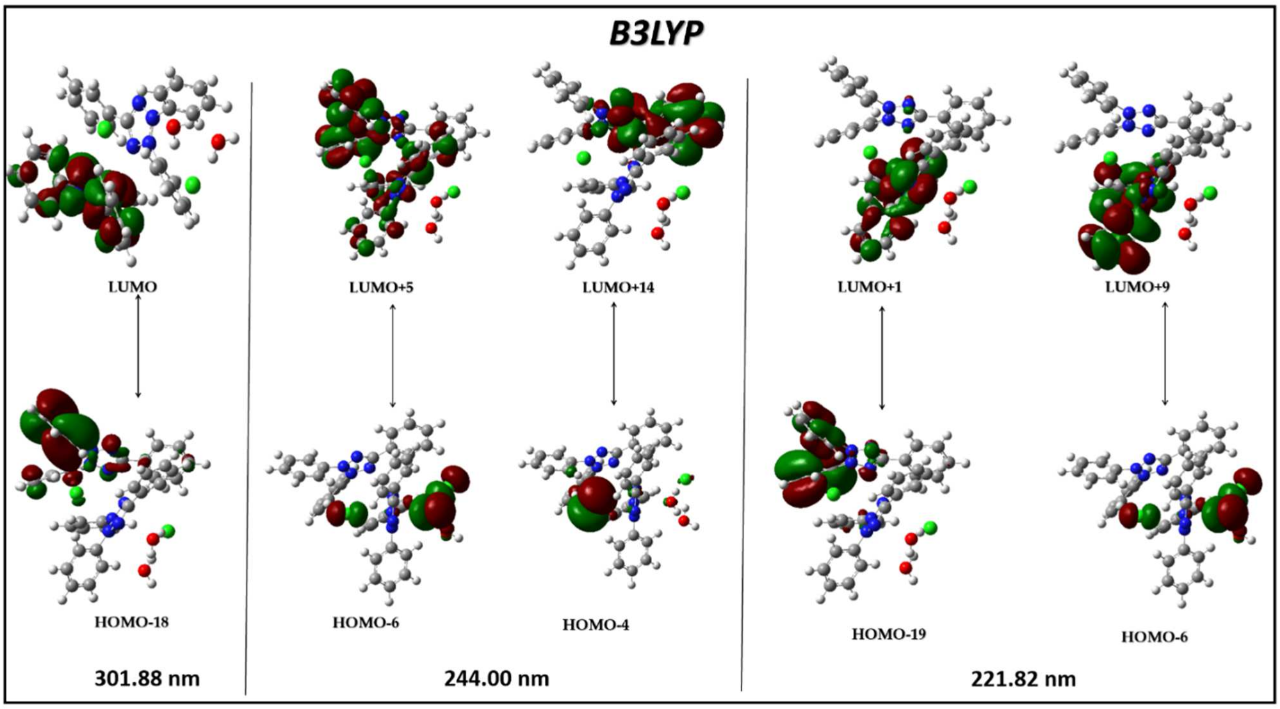

Figure S11. DFT-calculated molecular orbitals and associated TD-DFT-calculated electronic transitions for [TPT]Cl·H_2O using the B3LYP functional in the gas phase.

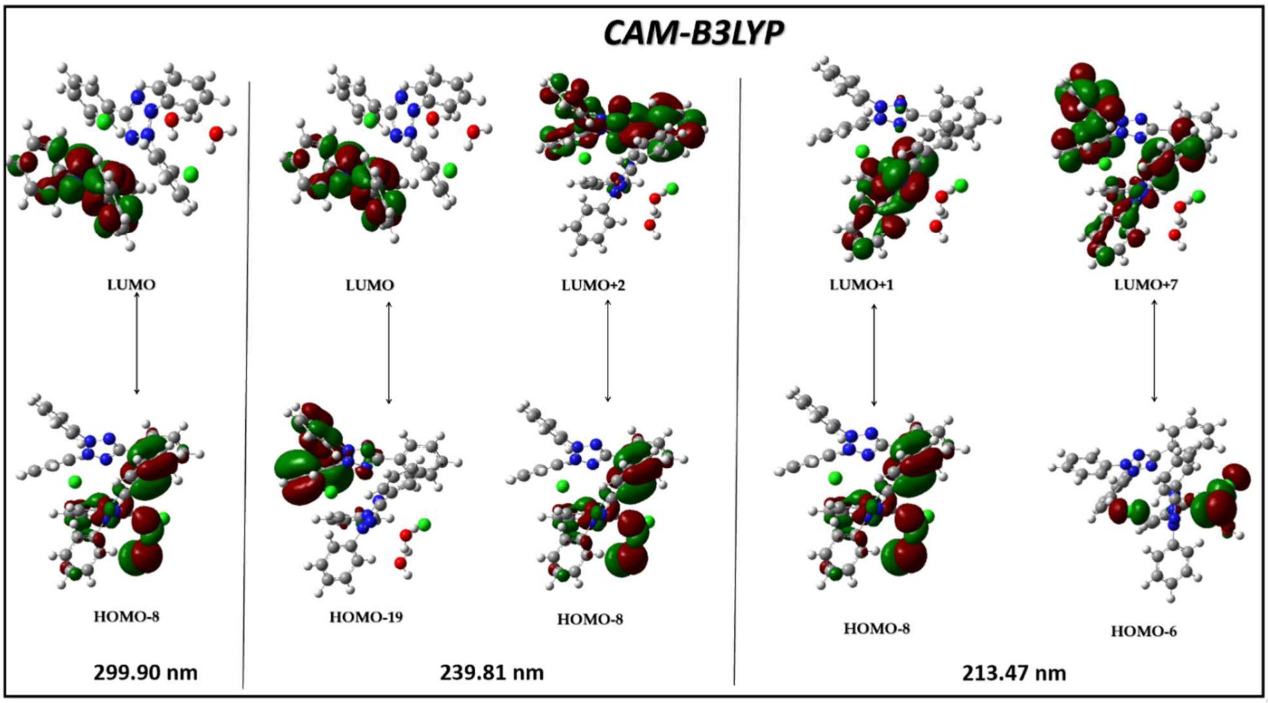

Figure S12. DFT-calculated molecular orbitals and associated TD-DFT-calculated electronic transitions for [TPT]Cl·H_2O using CAM-B3LYP functional in the gas phase.

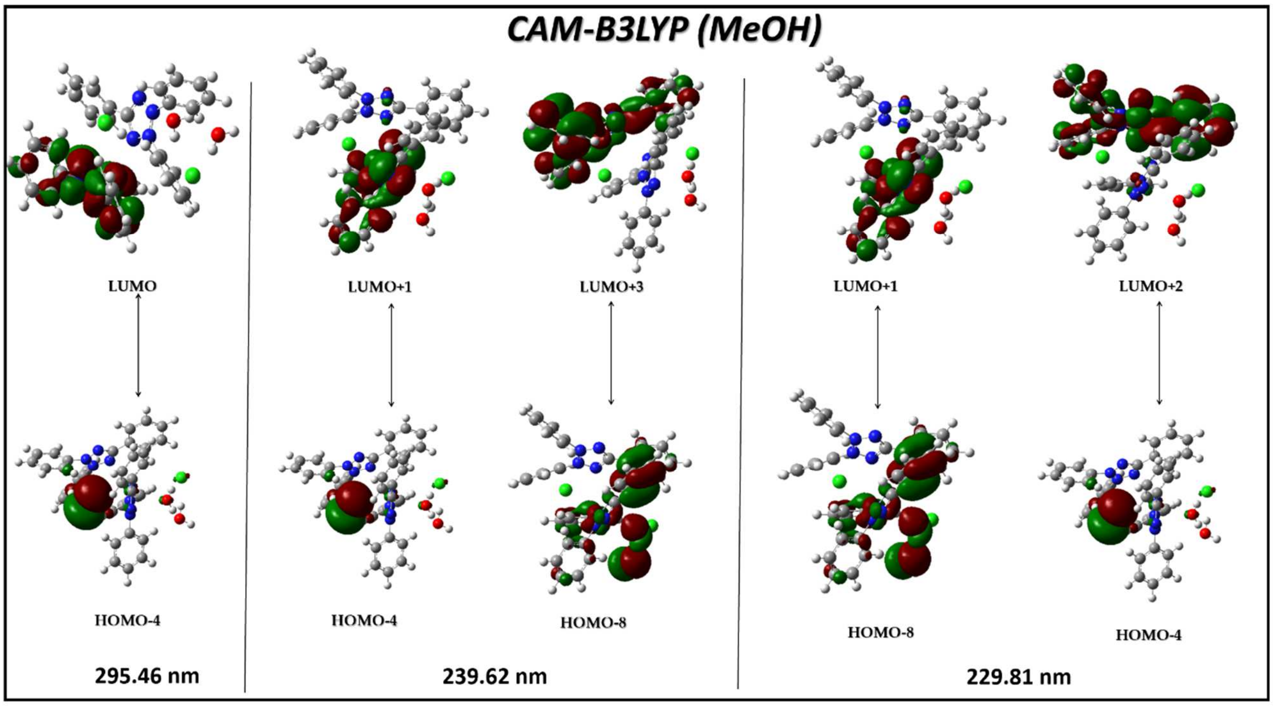

Figure S13. DFT-calculated molecular orbitals and associated TD-DFT-calculated electronic transitions for [TPT]Cl·H_2O using the CAM-B3LYP functional in MeOH (PCM).

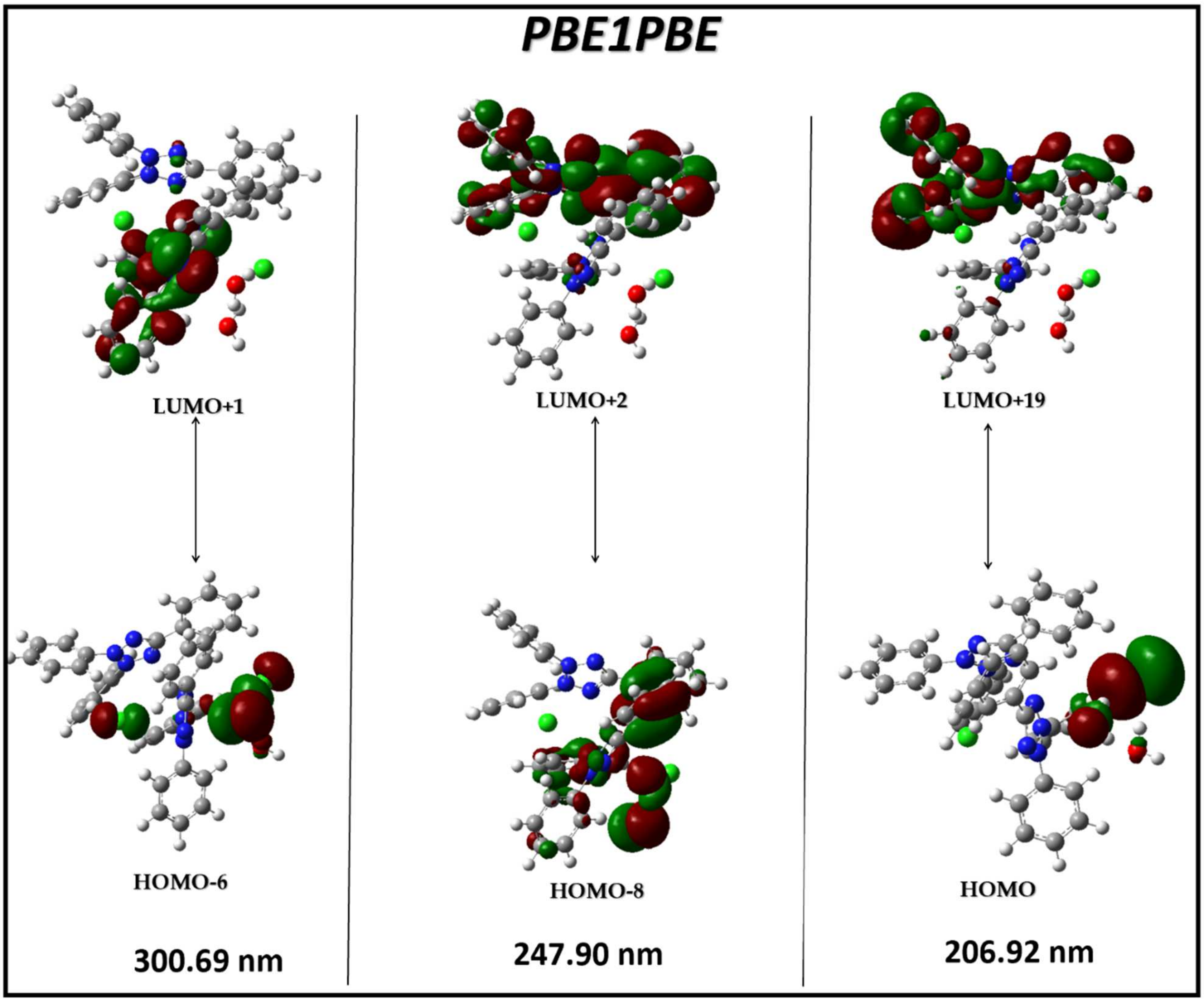

Figure S14. DFT-calculated molecular orbitals and associated TD-DFT-calculated electronic transitions for [TPT]Cl·H_2O using the PBE1PBE functional in the gas phase.

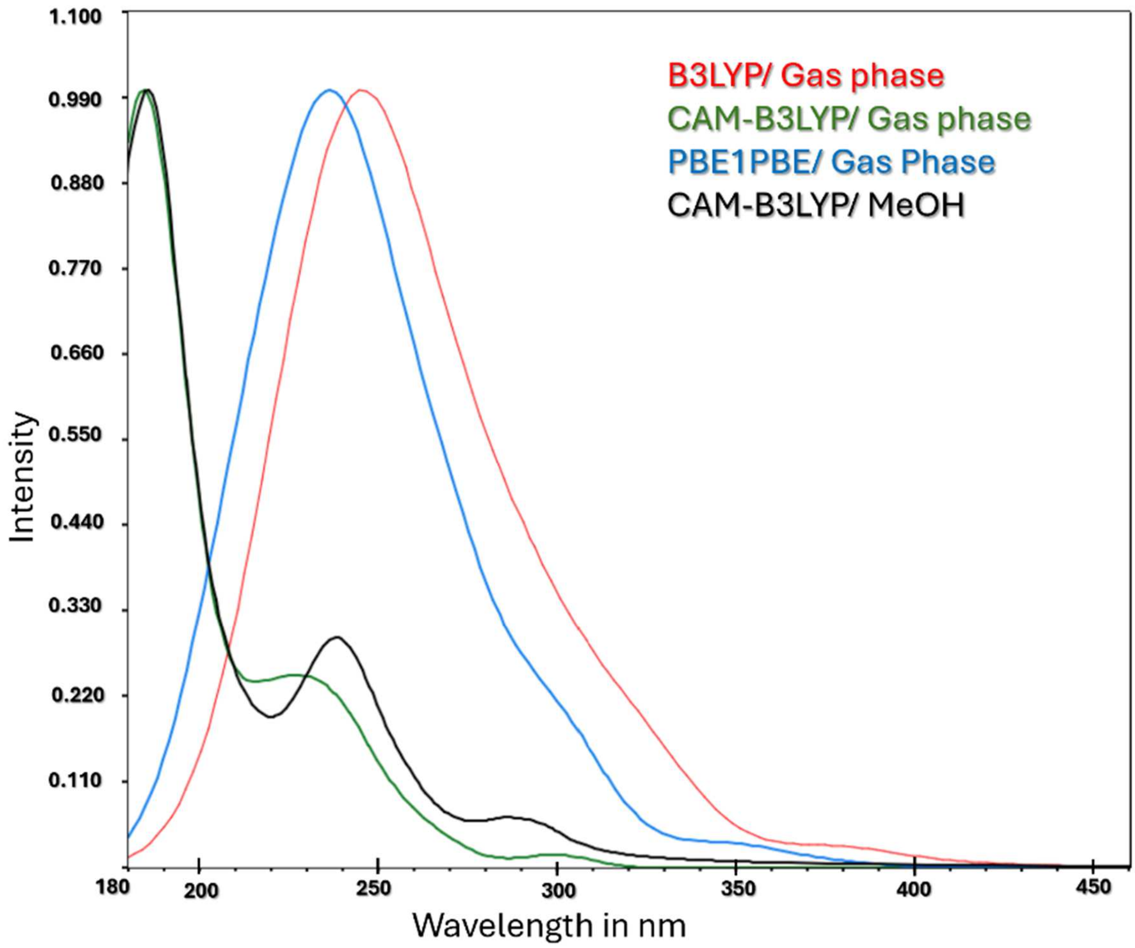

Figure S15. TD-DFT-calculated UV-vis absorption spectra on B3LYP, CAM-B3LYP, and PBE0 level of theory in gas phase and CAM-B3LYP in MeOH (PCM model).

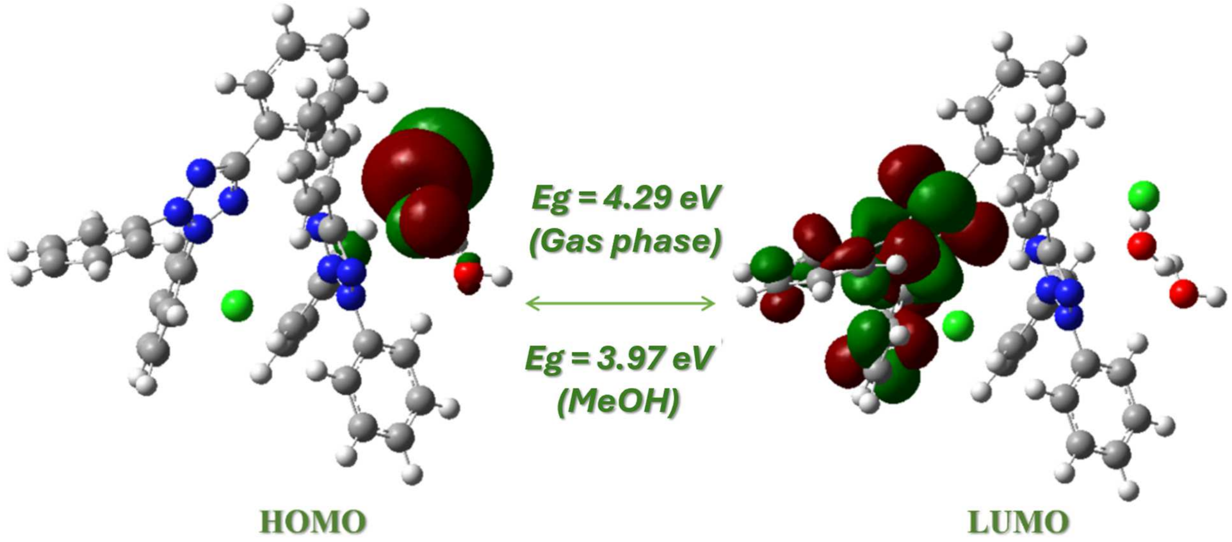

Figure S16. DFT-calculated frontier molecular orbitals and energy gap (*E*g) for [TPT]Cl·H_2O using the CAM-B3LYP functional in the gas phase and in MeOH using the PCM method.

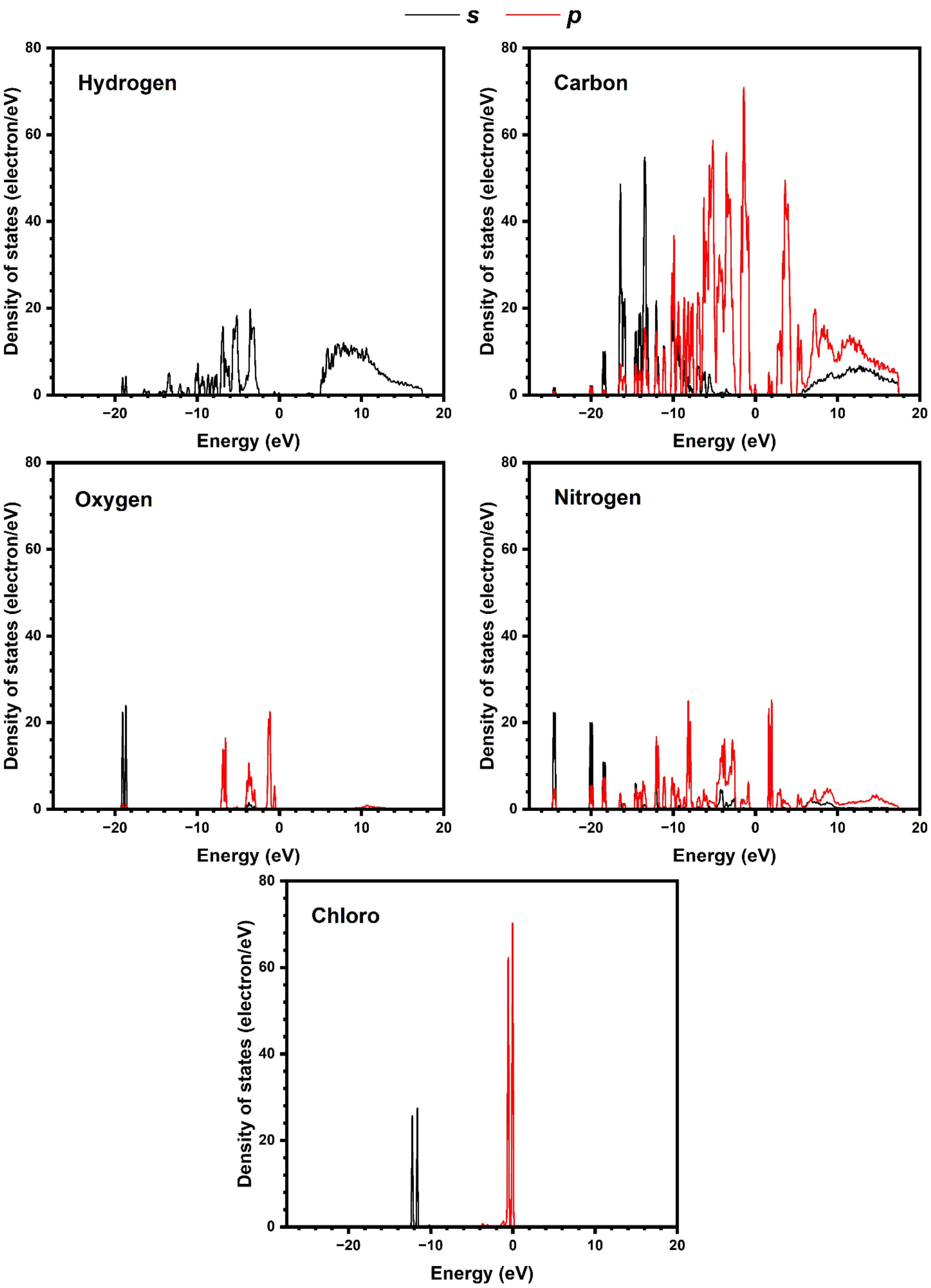

Figure S17. Partial density of states (PDOS) for atoms in [TPT]Cl·H_2O using the GGA+TS functional in the crystalline phase.

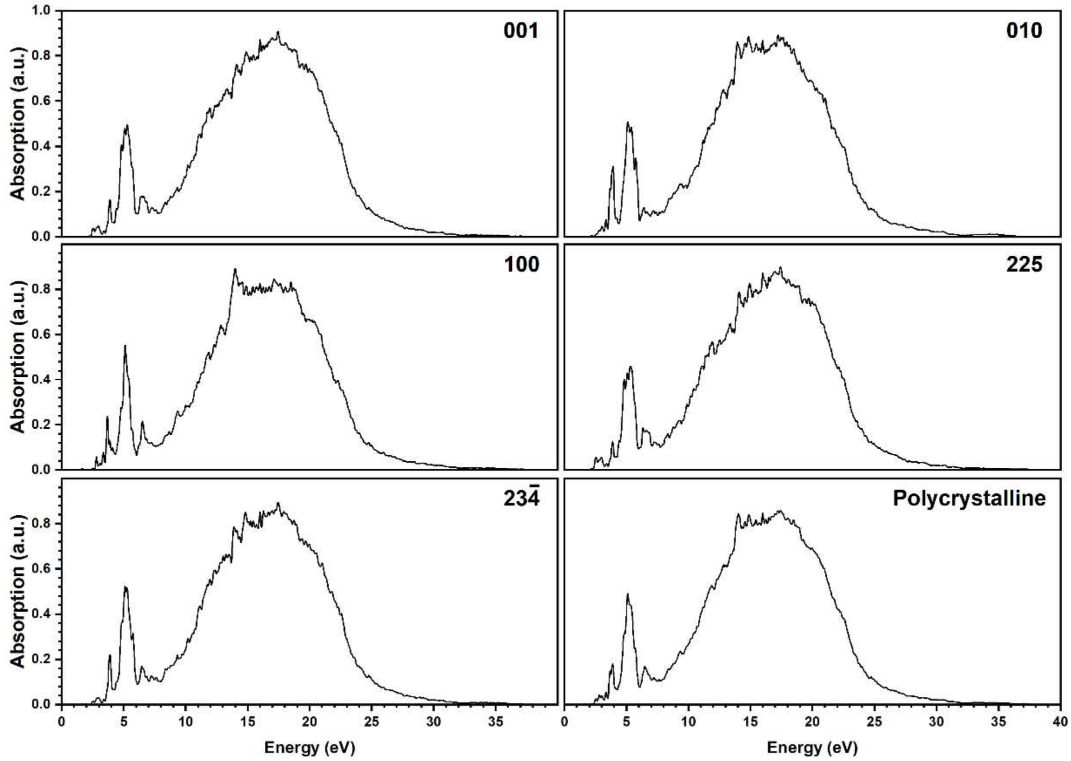

Figure S18. Absorption profiles in different directions of plane polarized light for [TPT]Cl.H_2O using the GGA+TS functional in the crystalline phase. The directions 225 and 23$\bar{4}$ are corresponding to molecular planes defined by the tetrazole and phenyl rings.

Supplementary Tables

Table S1. Selected crystallographic data of [TZT]Cl·H_2O and derivatives.[a]

Determination	This work	Lyssenko 2005	Fronczek 2024
Formula (M_W)	[TPT]Cl·H_2O (352.81)	[TPT]Cl·H_2O (352.81)	[TPT]Cl·2.5H_2O (379.84)
Year	2024	2005	2023
Publication	This work	Ref. 31	Ref. 32
CCDC	2373758	289399	2314756
CCDC code	TPT Cl H_2O	LAWYAK	ROJSUI
T (K)	100	220	100
device	Bruker D8 Venture	Synthex $P2_1$	Bruker D8 Venture
space group	$P\bar{1}$	$P\bar{1}$	$P\bar{1}$
Cell a (Å)	9.1013(7)	9.229(2)	9.5599(4)
b (Å)	12.1019(9)	12.207(2)	11.8056(5)
c (Å)	16.7963(12)	17.008(3)	17.5322(7)
α (°)	101.259(3)	100.79(3)	94.808(2)
β (°)	97.392(3)	98.64(3)	104.562(2)
γ (°)	103.926(3)	103.25(3)	95.408(2)
V (Å^3) / Z	1730.8(2) / 4	1794.3(6) / 4	1894.7(1) / 4
δ (g cm^{-3})	1.354	1.306	1.332
R_1 refined (all data)	0.0362	0.1325	0.0780
wR_2 refined (all data)	0.0923	0.1514	0.1413
Residual e/holes	0.42 / –0.50	0.31 / –0.45	0.63 / –0.67
Cl···N distances (Å)	Cl1: 3.165(1), 3.264(1), Cl2: 3.366(1), 3.412(1)	Cl1: 3.213(5), 3.303(5) Cl2: 3.418(5), 3.457(4)	Cl1: 3.813(1), 3.944(1) Cl2: 5.609(1), 6.725(1)
Cl···H distances (Å)	2.25(3), 2.38(2), 2.41(3)	2.366(2), 3.083(2)	2.30(1), 2.32(1), 2.34(1), 2.35(1)
Cl···O distances (Å)	3.222(1) to 3.255(1)	3.172(3) to 3.310(3)	3.130(1) to 3.201(1)

[a] From single crystal X-ray diffraction.

Table S2. Selected X–H···X and Cl···π distances (Å) of [TZT]Cl·H_2O.[a]

D–H···A	D–H (Å)	H···A (Å)	D···A (Å)	D–H···A (°)
O1–H1[a]···Cl2	0.85(3)	2.41(3)	3.22(1)	160.0(3)
O2–H2[a]···Cl2	0.96(2)	2.38(2)	3.26(1)	152.0(2)
O1–H1[b]···O2	0.89(3)	2.00(3)	2.87(1)	163.0(2)
C9′–H9′···O1	0.95(1)	2.57(2)	3.37(2)	142.4(1)
C4–H4···Cl2	0.95(2)	2.85(1)	3.76(1)	161.1(1)
C13–H13···Cl1	0.95(2)	2.78(1)	3.56(1)	140.1(1)
C15–H15···Cl1	0.95(2)	2.77(1)	3.56(1)	140.7(1)
C13′–H13′···Cl1	0.95(2)	3.09(1)	3.57(1)	113.1(1)
C15′–H15′···Cl1	0.95(2)	3.01(1)	3.63(1)	124.2(1)
Cl1···centroid (C1,N1,N2,N3,N4)	-	3.353(1)	-	-
Cl1···centroid (C1′,N1′,N2′,N3′,N4′)	-	3.164(1)	-	-

[a] From single crystal X-ray diffraction. Symmetry codes: −x+1, −y+1, −z+2; x−1, y−1, z.

Table S3. Selected experimental and DFT-calculated equilibrium distances for [TPT]Cl·H_2O.[a]

distances (Å)	exp	calculated					
method	sc-XRD	GGA (TS) [c]	B3LYP 6-311+G*	CAM-B3LYP 6-311+G*	PBE1PBE 6-311+G*	CAM-B3LYP 6-311+G*	CAM-B3LYP PecG-2
medium	MeOH [b]	solid state	gas phase	gas phase	gas phase	MeOH	gas phase
Cl1···N1′	3.494(1)	3.637	3.395	3.359	3.377	3.447	3.366
Cl1···N2′	3.264(1)	3.387	3.244	3.244	3.236	3.281	3.245
Cl1···N3′	3.165(1)	3.281	3.225	3.245	3.208	3.267	3.239
Cl1···N4′	3.357(1)	3.501	3.343	3.341	3.311	3.402	3.371
Cl1···N1	3.549(1)	3.635	3.980	4.007	3.993	3.679	3.836
Cl1···N2	3.372(1)	3.434	3.438	3.477	3.447	3.350	3.351
Cl1···N3	3.429(1)	3.587	3.587	3.598	3.604	3.455	3.450
Cl2···C1	3.458(1)	3.589	3.804	3.382	3.383	3.483	4.414
Cl2···H1a	2.410(3)	2.332	2.352	2.332	2.335	2.460	2.244
Cl2···H2a	2.380(2)	2.292	2.151	2.143	2.126	2.234	2.129
O2···H1b	2.000(3)	1.860	2.017	1.998	1.990	1.873	2.078

[a] The entries marked in grey represent the "wet pocket" of the structure. Experimental data from single crystal X-ray diffraction. [b] Crystallized from MeOH. [c] Using the CASTEP code on GGA (PBE) level theory including the Tkatchenko-Scheffler (TS) scheme. More data in Table S4.

Table S4. Selected experimental and calculated distances (Å) and angles (°) of [TZT]Cl·H_2O using CAM-B3LYP (MeOH).[a]

	experimental	calculated			experimental	calculated	
	XRD[1]	solid state	CAM-B3LYP (MeOH)	distances (Å)	XRD[2]	solid state	CAM-B3LYP (MeOH)
**distances (Å)**							
Cl1···N1	3.549(1)	3.635	4.007	Cl2···N1	3.504(1)	3.628	-
Cl1···N2	3.372(1)	3.434	3.477	Cl2···N2	3.412(1)	3.532	-
Cl1···N3	3.429(1)	3.587	3.598	Cl2···N3	3.366(1)	3.488	-
Cl1···N4	3.648(1)	3.762	3.863	Cl2···N4	3.418(1)	3.547	-
Cl1···C1	3.685(1)	3.804	3.959	Cl2···C1	3.458(1)	3.589	3.483
Cl1···N1′	3.494(1)	3.637	3.447	Cl2···H1[a]	2.410(3)	2.332	2.460
Cl1···N2′	3.264(1)	3.387	3.281	Cl2···H2[a]	2.380(2)	2.292	2.234
Cl1···N3′	3.165(1)	3.281	3.267	Cl2···H2[b]	2.250(3)	2.118	3.678
Cl1···N4′	3.357(1)	3.501	3.402	O2···H1[b]	2.000(3)	1.860	1.873
Cl1···C1′	3.508(1)	3.664	3.483				
C1–C2	1.463(2)	1.457	1.455	C1′–C2′	1.460(2)	1.456	1.454
C1–N1	1.345(2)	1.360	1.342	C1′–N1′	1.344(2)	1.359	1.339
C1–N4	1.346(2)	1.360	1.342	C1′–N4′	1.349(1)	1.360	1.339
N1–N2	1.308(2)	1.329	1.297	N1′–N2′	1.315(2)	1.338	1.293
N2–N3	1.335(2)	1.374	1.329	N2′–N3′	1.339(1)	1.380	1.326
N3–N4	1.308(2)	1.331	1.296	N3′–N4′	1.304(2)	1.329	1.293
C8–N2	1.448(2)	1.448	1.429	C8′–N2′	1.445(2)	1.438	1.428
C14–N3	1.446(2)	1.446	1.431	C14′–N3′	1.443(2)	1.443	1.427
**angles (°)**				**angles (°)**			
C1–N4–N3	103.5(1)	104.1	104.3	C1′–N4′–N3′	103.8(1)	104.4	104.1
N2–N3–N4	110.2(1)	109.5	109.9	N2′–N3′–N4′	110.3(1)	109.4	110.0
C2–C1–N1	123.7(1)	123.4	124.2	C2′–C1′–N1′	124.1(1)	123.8	124.3
N1–N2–C8	123.3(1)	123.8	124.3	N1′–N2′–C8′	123.0(1)	124.1	124.7
N4–N3–C14	123.1(1)	123.7	124.3	N4′–N3′–C14′	123.9(1)	123.2	124.6
Cl1–N2–N3	81.1(1)	81.7	83.2	Cl1′–N2′–N3′	73.9(1)	73.7	-
Cl1–N3–N2	76.3(1)	75.5	74.3	Cl1′–N3′–N2′	82.2(1)	82.4	-
Cl2–H1[a]–O1	160.0(3)	148.7	144.5				
Cl2–H2[a]–O2	152.0(2)	158.5	158.9				
O2–H1[b]–O1	163.0(2)	159.5	153.9				

[a] Two independent cations and anions in the unit cell.

Table S5. Experimental and DFT-optimized unit cell parameters, volume, and density of $[TPT]Cl{\cdot}H_2O$ in the solid state.[a]

	a (Å)	Δa (Å)	b (Å)	Δb (Å)	c (Å)	Δc (Å)	V (Å^3)	ΔV (Å^3)
Exp	9.1013(7)	-	12.1019(9)	-	16.7963(12)	-	1730.8(2)	-
Calc.	9.0989	0.0024	12.1123	–0.0104	16.8541	–0.0578	1743.16	–12.34
	α (°)	$\Delta\alpha$ (°)	β (°)	$\Delta\beta$ (°)	γ (°)	$\Delta\gamma$ (°)	δ (g cm^{-3})	$\Delta\delta$
Exp	101.259(3)	-	97.392(3)	-	103.926(3)	-	1.354	-
Calc.	101.537	–0.278	96.377	–1.985	103.738	0.188	1.344	–0.01

[a] Experimental data from single-crystal X-ray diffraction (see Table S1). Calculations using GGA+TS830eV.

Table S6. DFT-calculated HOMO and LUMO energies for $[TPT]Cl{\cdot}H_2O$ and [TPT]Cl.

	**[TPT]Cl·H₂O**				**[TPT]Cl**			
**method**	**B3LYP**	**PBE1PBE**	**CAM-B3LYP**	**CAM-B3LYP**	**APFD/6-311**	**++G(2d,p)**	**PM3-based**	**Hartree-Fock**
**medium**	gas phase	gas phase	gas phase	MeOH	gas phase	MeOH	gas phase	water
**reference**	this work	this work	this work	this work	[1]	[1]	[2]	[2]
**E_{HOMO} (eV)**	–4.69	–4.94	–6.32	–6.94	–10.13	–7.59	–9.32	–9.29
**E_{LUMO} (eV)**	–3.30	–3.09	–2.03	–2.97	–6.22	–3.22	–0.29	–0.27
**Eg (eV) [a]**	1.39	1.85	4.29	3.97	3.90	4.37	9.03	9.02

[a] Energy Gap $Eg = E_{LUMO} – E_{HOMO}$.

Table S7. DFT-calculated global reactivity descriptors for $[TPT]Cl{\cdot}H_2O$ and [TPT]Cl.[a.]

	**[TPT]Cl·H₂O**				**[TPT]Cl**	
**functional**	**B3LYP**	**PBE1PBE**	**CAM-B3LYP**	**CAM-B3LYP**		
medium	gas phase	gas phase	gas phase	MeOH	gas phase	MeOH
reference	this work	this work	this work	this work	26	26
I (eV)	4.69	4.94	6.32	6.94	10.13	7.59
A (eV)	3.30	3.09	2.03	2.97	6.22	3.26
η (eV)	0.69	0.92	2.15	1.99	1.95	2.17
μ (eV)	–3.99	–4.02	–4.18	–4.96	–8.17	–5.41
ω (eV)	11.50	8.70	4.10	6.19	17.10	6.77
χ (eV)	3.99	4.02	4.18	4.96	8.17	5.41
S (eV^{-1})	0.72	0.54	0.23	0.25	0.26	0.23

[a] Energy Gap $Eg = E_{LUMO}–E_{HOMO}$; Ionization Energy $I = −E_{HOMO}$; Electron Affinity $A = −E_{LUMO}$; Chemical Hardness $\eta = \frac{(E_{LUMO}-E_{HOMO})}{2}$; Chemical Potential $\mu = \frac{(E_{HOMO}+E_{LUMO})}{2}$; Electrophilicity $\omega = \frac{\mu^2}{2\eta}$; Electronegativity $\chi = \frac{(I+A)}{2}$; Softness $S = \frac{1}{2\eta}$.

Table S8. XYZ coordinates of the DFT-optimized structure of $[TPT]Cl{\cdot}H_2O$ using the B3LYP functional.

Cl	0.950343	-1.248048	6.455794
Cl	2.498348	3.188737	0.396942
O	2.476363	3.628486	3.595199
H	2.147585	3.642089	2.677688
H	3.428617	3.764212	3.475204
O	4.968726	3.208751	2.297211
H	4.300304	3.160726	1.570690
H	5.642421	3.829026	2.000398
N	-1.923211	-1.012976	3.711740
N	-1.291641	-2.120247	4.000113
N	-0.211236	-2.243528	3.211074
N	-0.149273	-1.232559	2.385304
C	-1.212475	-0.465259	2.704259
C	-1.574506	0.770811	2.026231
C	-0.628414	1.455524	1.255304

H	0.384571	1.088765	1.163645
C	-0.974315	2.650285	0.635005
H	-0.210568	3.182888	0.080480
C	-2.268532	3.152786	0.770377
H	-2.537001	4.086355	0.287140
C	-3.215028	2.467789	1.533960
H	-4.219060	2.863335	1.643012
C	-2.870813	1.282274	2.170100
H	-3.593772	0.757548	2.783231
C	-1.775925	-3.099860	4.935119
C	-2.081911	-4.372824	4.463252
H	-1.940241	-4.625396	3.419275
C	-2.570906	-5.309538	5.366026
H	-2.815957	-6.308041	5.023270
C	-2.750582	-4.958030	6.704631
H	-3.130220	-5.692858	7.406102
C	-2.444663	-3.672275	7.146110
H	-2.573576	-3.406874	8.188724
C	-1.947413	-2.721275	6.259325
H	-1.640806	-1.736001	6.581492
C	0.702620	-3.349597	3.192726
C	1.184893	-3.871082	4.387914
H	0.937817	-3.393794	5.331160
C	2.061024	-4.948956	4.315650
H	2.459523	-5.365387	5.233612
C	2.444250	-5.471145	3.081083
H	3.130554	-6.309958	3.038515
C	1.963132	-4.910066	1.898078
H	2.274527	-5.304218	0.937769
C	1.081587	-3.837008	1.945225
H	0.704099	-3.375059	1.041935
N	1.767972	1.964738	6.023956
N	2.668369	1.267667	5.397315
N	2.170982	0.793593	4.250608
N	0.943804	1.200589	4.104463
C	0.701378	1.924642	5.209266
C	-0.570623	2.576471	5.503522
C	-1.063708	2.561619	6.812571
H	-0.502889	2.049915	7.585894
C	-2.274381	3.182153	7.098685
H	-2.661130	3.167505	8.111978
C	-2.990157	3.819328	6.084540
H	-3.932347	4.307483	6.311439
C	-2.494524	3.833565	4.782131
H	-3.044079	4.333351	3.992805
C	-1.286311	3.211682	4.486062
H	-0.886142	3.237092	3.480675
C	2.864705	-0.106715	3.372710
C	3.021719	0.258486	2.044975
H	2.664786	1.212455	1.667557
C	3.698743	-0.619269	1.203017
H	3.839596	-0.339293	0.165356
C	4.204748	-1.819096	1.698559
H	4.739948	-2.494344	1.038841
C	4.030321	-2.158185	3.041067
H	4.410867	-3.098514	3.421355
C	3.347197	-1.303335	3.897106
H	3.152097	-1.556432	4.931372
C	4.003454	1.034883	5.863897
C	4.152594	0.391821	7.086811

H	3.272917	0.057075	7.623132
C	5.445353	0.159830	7.545494
H	5.594196	-0.343604	8.494060
C	6.542114	0.561129	6.782002
H	7.547388	0.373760	7.144307
C	6.356196	1.200299	5.556396
H	7.210200	1.513326	4.966777
C	5.073103	1.450524	5.078172
H	4.921725	1.952820	4.128235

Table S9. XYZ coordinates of the DFT-optimized structure of [TPT]Cl·H_2O using the PBE1PBE functional.

Cl	0.978795	-1.233189	6.461203
Cl	2.504362	3.165450	0.392786
O	2.485491	3.584459	3.573330
H	2.151962	3.597210	2.660830
H	3.431808	3.736695	3.446913
O	4.955158	3.226080	2.272785
H	4.286994	3.165712	1.550515
H	5.610218	3.861099	1.976935
N	-1.913120	-0.985578	3.719013
N	-1.289111	-2.085949	4.009270
N	-0.229706	-2.229250	3.215554
N	-0.165865	-1.239767	2.378141
C	-1.212833	-0.461548	2.699531
C	-1.569810	0.767898	2.013139
C	-0.620614	1.444912	1.245978
H	0.394061	1.074867	1.160557
C	-0.961375	2.634962	0.619897
H	-0.192828	3.164034	0.065502
C	-2.252818	3.138010	0.746838
H	-2.518268	4.070123	0.257166
C	-3.202123	2.459207	1.506910
H	-4.207629	2.855798	1.607500
C	-2.863080	1.278562	2.148929
H	-3.589819	0.755354	2.761344
C	-1.768124	-3.043817	4.959409
C	-2.085965	-4.319335	4.511579
H	-1.952611	-4.591531	3.470004
C	-2.576227	-5.233464	5.431626
H	-2.831251	-6.236642	5.107748
C	-2.746071	-4.857191	6.761182
H	-3.128398	-5.576909	7.478011
C	-2.429440	-3.569291	7.177650
H	-2.552066	-3.284115	8.216662
C	-1.930440	-2.639986	6.274246
H	-1.616496	-1.648509	6.576123
C	0.669653	-3.339386	3.204452
C	1.157325	-3.846759	4.400286
H	0.924865	-3.350085	5.341075
C	2.019066	-4.932836	4.333492
H	2.422253	-5.341717	5.253876
C	2.383215	-5.474174	3.104957
H	3.059962	-6.322113	3.067043
C	1.898642	-4.924930	1.921510
H	2.196623	-5.335873	0.962971
C	1.030346	-3.844691	1.962111
H	0.647218	-3.389872	1.055879
N	1.784316	1.945911	6.003036

N	2.679004	1.260739	5.372780
N	2.185297	0.785664	4.238767
N	0.961511	1.175693	4.094672
C	0.718542	1.893640	5.196882
C	-0.559593	2.520823	5.503258
C	-1.051909	2.459758	6.807221
H	-0.482732	1.931842	7.565349
C	-2.269456	3.052430	7.108224
H	-2.657883	3.002864	8.120642
C	-2.990906	3.706636	6.113724
H	-3.941128	4.174701	6.353101
C	-2.495370	3.765973	4.815978
H	-3.051533	4.281670	4.040067
C	-1.280254	3.171819	4.504459
H	-0.878125	3.230628	3.498813
C	2.880799	-0.108651	3.366116
C	3.044443	0.257694	2.042281
H	2.691943	1.216177	1.662505
C	3.720351	-0.621039	1.205435
H	3.867293	-0.340676	0.167633
C	4.217774	-1.820734	1.701453
H	4.754670	-2.497460	1.043118
C	4.036527	-2.160094	3.039787
H	4.414572	-3.102134	3.422141
C	3.354372	-1.304803	3.891952
H	3.152690	-1.555492	4.928347
C	4.012887	1.040784	5.829603
C	4.170478	0.408955	7.053961
H	3.292677	0.071898	7.595455
C	5.463738	0.191583	7.508299
H	5.620438	-0.304269	8.460512
C	6.551333	0.596259	6.739660
H	7.560259	0.419944	7.099855
C	6.356581	1.224493	5.513594
H	7.206532	1.541834	4.918876
C	5.073127	1.460886	5.038645
H	4.914247	1.959666	4.085545

Table S10. XYZ coordinates of the DFT-optimized structure of [TPT]Cl·H_2O using the CAM-B3LYP functional in the gas phase.

Cl	0.994611	-1.214195	6.471223
Cl	2.537701	3.218856	0.352094
O	2.423640	3.602786	3.534431
H	2.116886	3.614400	2.611286
H	3.359170	3.835589	3.449559
O	4.942521	3.412783	2.306563
H	4.304334	3.321122	1.561083
H	5.608102	4.044879	2.022744
N	-1.920204	-1.031314	3.727622
N	-1.303790	-2.133620	4.029497
N	-0.230380	-2.273964	3.257949
N	-0.150448	-1.279050	2.428313
C	-1.202157	-0.501930	2.726890
C	-1.551146	0.729174	2.029319
C	-0.597459	1.397333	1.265129
H	0.414171	1.022545	1.189519
C	-0.931650	2.579270	0.624271
H	-0.162329	3.101929	0.068523
C	-2.221543	3.083717	0.736625

H	-2.481847	4.009757	0.236174
C	-3.175567	2.414195	1.494657
H	-4.179763	2.812504	1.584267
C	-2.842691	1.240280	2.148411
H	-3.574704	0.724436	2.757901
C	-1.804072	-3.100797	4.971868
C	-2.193285	-4.341860	4.492835
H	-2.100633	-4.584294	3.441076
C	-2.700580	-5.262253	5.394202
H	-3.009996	-6.240258	5.046533
C	-2.816718	-4.923718	6.737740
H	-3.213293	-5.647389	7.440483
C	-2.427949	-3.668957	7.186031
H	-2.509839	-3.413727	8.235280
C	-1.911037	-2.734937	6.299885
H	-1.542687	-1.771795	6.626053
C	0.664408	-3.398833	3.247737
C	1.173506	-3.887871	4.437999
H	0.959512	-3.382336	5.374325
C	2.026710	-4.978929	4.372359
H	2.445350	-5.375716	5.289295
C	2.361201	-5.541435	3.147605
H	3.032492	-6.391727	3.109661
C	1.852448	-5.012209	1.967479
H	2.126196	-5.441415	1.011439
C	0.991817	-3.928310	2.009477
H	0.591742	-3.489258	1.104621
N	1.800064	1.992096	5.985155
N	2.700790	1.311079	5.357177
N	2.200372	0.808473	4.239764
N	0.967218	1.174288	4.110209
C	0.724818	1.907434	5.199137
C	-0.564757	2.518846	5.510841
C	-1.072554	2.415191	6.803155
H	-0.511467	1.868993	7.552034
C	-2.296066	2.990645	7.105784
H	-2.696597	2.908767	8.109760
C	-3.007922	3.668743	6.123283
H	-3.963131	4.122521	6.363630
C	-2.497200	3.769565	4.836348
H	-3.047347	4.302489	4.069987
C	-1.275324	3.193576	4.524693
H	-0.862578	3.282312	3.527532
C	2.903965	-0.081531	3.356473
C	3.089492	0.307779	2.045260
H	2.747752	1.270731	1.678341
C	3.763146	-0.561222	1.199270
H	3.923751	-0.264167	0.169871
C	4.236910	-1.775388	1.676670
H	4.770906	-2.445670	1.011994
C	4.035674	-2.137803	3.004087
H	4.397645	-3.090229	3.371608
C	3.356056	-1.290517	3.863151
H	3.145020	-1.557067	4.891156
C	4.048738	1.116058	5.807472
C	4.228802	0.451300	7.008006
H	3.365844	0.074311	7.543768
C	5.527419	0.259343	7.452796
H	5.701893	-0.259820	8.387614
C	6.598399	0.721072	6.696442

H	7.611872	0.564425	7.048408
C	6.381555	1.380805	5.493293
H	7.218770	1.740840	4.907628
C	5.091082	1.592198	5.030103
H	4.915681	2.113613	4.094271

Table S11. XYZ coordinates of the DFT-optimized structure of [TPT]Cl·H_2O using CAM-B3LYP in MeOH.

Cl	0.628053	-1.024125	6.257406
Cl	2.630455	3.874789	0.539188
O	2.926973	3.902460	3.821611
H	2.458938	4.045578	2.986920
H	3.842013	3.753567	3.531645
O	5.149348	3.172544	2.322718
H	4.532092	3.343569	1.584869
H	5.923690	3.720532	2.171524
N	-2.013665	-1.394732	3.723023
N	-1.346378	-2.477208	3.974610
N	-0.230085	-2.500906	3.253584
N	-0.165240	-1.447278	2.500379
C	-1.278750	-0.759271	2.797361
C	-1.665960	0.488160	2.156123
C	-0.768156	1.142897	1.310863
H	0.220709	0.729712	1.149353
C	-1.139057	2.329748	0.696945
H	-0.428041	2.847524	0.061795
C	-2.409861	2.858190	0.912615
H	-2.698720	3.787022	0.431371
C	-3.307206	2.201409	1.749775
H	-4.296370	2.613243	1.919469
C	-2.937498	1.020610	2.378246
H	-3.631588	0.510429	3.037226
C	-1.795591	-3.515067	4.851009
C	-1.946807	-4.799968	4.346303
H	-1.723231	-5.022782	3.309299
C	-2.402601	-5.788209	5.206891
H	-2.530067	-6.799675	4.838028
C	-2.704819	-5.477914	6.529971
H	-3.061442	-6.256262	7.196232
C	-2.556582	-4.177747	7.003368
H	-2.791897	-3.941405	8.035126
C	-2.094171	-3.175398	6.161412
H	-1.928153	-2.162253	6.505694
C	0.743862	-3.546722	3.261653
C	1.291616	-3.954031	4.469768
H	1.008151	-3.454356	5.390415
C	2.239652	-4.967334	4.440300
H	2.690728	-5.301383	5.368057
C	2.621630	-5.538294	3.229297
H	3.365044	-6.328326	3.217436
C	2.064421	-5.097386	2.032255
H	2.368774	-5.539631	1.090302
C	1.111841	-4.088546	2.038384
H	0.659950	-3.730316	1.120732
N	1.802226	2.160549	6.031753

N	2.634525	1.404314	5.392879
N	2.129812	1.060170	4.216126
N	0.968655	1.605808	4.054822
C	0.768615	2.288193	5.189881
C	-0.422944	3.074325	5.467050
C	-0.706566	3.479265	6.773037
H	-0.039902	3.200681	7.582158
C	-1.846124	4.228620	7.028192
H	-2.068932	4.540125	8.043340
C	-2.701964	4.575938	5.986021
H	-3.592301	5.162563	6.188911
C	-2.416524	4.172205	4.685103
H	-3.080258	4.442581	3.870662
C	-1.279523	3.421930	4.421551
H	-1.048077	3.113510	3.408159
C	2.745783	0.171225	3.283888
C	2.890623	0.602183	1.975037
H	2.580731	1.599733	1.676953
C	3.471723	-0.271012	1.064932
H	3.599150	0.042846	0.034446
C	3.898804	-1.530179	1.475489
H	4.355104	-2.205656	0.759285
C	3.739718	-1.932914	2.798465
H	4.056485	-2.921481	3.111310
C	3.148845	-1.083144	3.721479
H	2.966463	-1.387368	4.745703
C	3.919930	1.018684	5.879618
C	3.977042	0.392648	7.115895
H	3.061203	0.185020	7.656824
C	5.223283	0.020829	7.601537
H	5.296967	-0.474370	8.563447
C	6.368432	0.272398	6.851265
H	7.339011	-0.024972	7.234290
C	6.278586	0.904661	5.614210
H	7.174418	1.106110	5.037194
C	5.043924	1.292045	5.111054
H	4.966491	1.794019	4.151689

Table S12. XYZ coordinates of the DFT-optimized structure of [TPT]Cl·H_2O using using the CAM-B3LYP functional and pecG-2 basis set in the gas phase.

Cl	0.902365	-1.205748	6.333805
Cl	2.382149	3.374006	0.523919
O	2.589448	3.763417	3.649852
H	2.255831	3.745807	2.733870
H	3.527754	3.928101	3.502140
O	5.002491	3.292887	2.183757
H	4.241553	3.294116	1.557847
H	5.647818	3.878329	1.784446
N	-1.950913	-1.138966	3.770085
N	-1.319056	-2.234407	4.045175
N	-0.242731	-2.335520	3.281191
N	-0.172122	-1.318193	2.483854
C	-1.237641	-0.568997	2.791483
C	-1.608483	0.665028	2.116532
C	-0.684062	1.335944	1.326570
H	0.323728	0.966735	1.225798

C	-1.040166	2.513660	0.699578
H	-0.289377	3.044159	0.131852
C	-2.324880	3.009800	0.848316
H	-2.602803	3.932312	0.358628
C	-3.250374	2.338166	1.631402
H	-4.249561	2.731314	1.750528
C	-2.894391	1.169632	2.273200
H	-3.603633	0.650180	2.900026
C	-1.799998	-3.226360	4.965405
C	-2.160649	-4.463002	4.465854
H	-2.063324	-4.681642	3.412933
C	-2.646000	-5.408292	5.347224
H	-2.932863	-6.383089	4.982731
C	-2.769148	-5.098320	6.692147
H	-3.148786	-5.840804	7.378940
C	-2.409458	-3.847263	7.162160
H	-2.499511	-3.614273	8.212362
C	-1.914260	-2.888975	6.296613
H	-1.566412	-1.925268	6.635446
C	0.671471	-3.439154	3.249675
C	1.203143	-3.928441	4.425127
H	0.986484	-3.433201	5.363332
C	2.075921	-4.997174	4.338520
H	2.512143	-5.394485	5.242815
C	2.406633	-5.538549	3.107978
H	3.092991	-6.371190	3.053786
C	1.874076	-5.010707	1.943026
H	2.143898	-5.424037	0.982912
C	0.994270	-3.948839	2.006570
H	0.574567	-3.510199	1.114503
N	1.817730	2.016114	5.998831
N	2.698446	1.318519	5.366905
N	2.201913	0.880925	4.225170
N	0.992198	1.304735	4.085124
C	0.758258	2.009590	5.191550
C	-0.499170	2.681483	5.490386
C	-0.986003	2.674031	6.790511
H	-0.435116	2.152843	7.559358
C	-2.175485	3.314086	7.077812
H	-2.559490	3.306249	8.087574
C	-2.875109	3.961619	6.072519
H	-3.803740	4.465327	6.300556
C	-2.385988	3.967272	4.777924
H	-2.926549	4.476171	3.993749
C	-1.198108	3.326635	4.481533
H	-0.802072	3.338629	3.477360
C	2.878451	-0.003958	3.321725
C	3.037202	0.398890	2.015156
H	2.689178	1.367411	1.671453
C	3.685352	-0.461677	1.147643
H	3.825652	-0.155475	0.121611
C	4.158391	-1.681339	1.597890
H	4.671619	-2.344833	0.916140
C	3.980247	-2.059476	2.919817
H	4.338395	-3.017519	3.265367
C	3.326797	-1.221046	3.800591
H	3.128481	-1.501566	4.823938
C	4.028427	1.069282	5.829824
C	4.171456	0.391494	7.023251
H	3.291777	0.043586	7.544877

C	5.453257	0.149423	7.479037
H	5.598151	-0.380785	8.408526
C	6.545476	0.575355	6.740460
H	7.545044	0.379378	7.101117
C	6.367471	1.248812	5.543555
H	7.220869	1.580336	4.971249
C	5.094484	1.509866	5.071087
H	4.943413	2.038655	4.139117

Table S13. XYZ coordinates of the DFT-optimized structure of [TPT]Cl·H_2O in the solid state at GGA+TS level of theory.

Cl1	0.15832	0.04220	0.38049
Cl2	0.33472	0.29204	-0.04007
O1	0.46801	0.39121	0.15228
H1a	0.39084	0.35672	0.10255
H1b	0.55668	0.42293	0.12699
O2	0.67248	0.45451	0.04593
H2a	0.58188	0.40437	0.00664
H2b	0.67203	0.53478	0.04153
N1	-0.18691	-0.06274	0.22616
N2	-0.14084	-0.15357	0.24188
N3	-0.02369	-0.16885	0.19965
N4	0.00537	-0.08832	0.15594
C1	-0.09669	-0.02456	0.17270
C2	-0.10897	0.07387	0.13735
C3	-0.00480	0.11429	0.08745
H3	0.08399	0.07040	0.07416
C4	-0.01425	0.21085	0.05607
H4	0.07009	0.24191	0.01877
C5	-0.12912	0.26666	0.07316
H5	-0.13897	0.34225	0.04920
C6	-0.23571	0.22464	0.12116
H6	-0.32719	0.26666	0.13294
C7	-0.22558	0.12889	0.15342
H7	-0.30745	0.09531	0.19083
C8	-0.20995	-0.22592	0.29381
C9	-0.30666	-0.33675	0.25698
H9	-0.32662	-0.36856	0.19044
C10	-0.38037	-0.40193	0.30778
H10	-0.45683	-0.48832	0.27943
C11	-0.35768	-0.35534	0.39248
H11	-0.41768	-0.40599	0.43148
C12	-0.25873	-0.24438	0.42782
H12	-0.23803	-0.20699	0.49404
C13	-0.18347	-0.17818	0.37817
H13	-0.10361	-0.09208	0.40299
C14	0.06183	-0.25479	0.20305
C15	0.16056	-0.24363	0.27542
H15	0.16551	-0.17398	0.32878
C16	0.25614	-0.31835	0.27425
H16	0.33553	-0.31087	0.32966

C17	0.25124	-0.40149	0.20255
H17	0.32739	-0.45885	0.20114
C18	0.14828	-0.41234	0.13153
H18	0.14548	-0.47713	0.07591
C19	0.05177	-0.33858	0.13114
H19	-0.03010	-0.34466	0.07721
N1'	0.40890	0.32836	0.38820
N2'	0.46390	0.24253	0.35046
N3'	0.37554	0.19095	0.27399
N4'	0.26686	0.24514	0.26195
C1'	0.28828	0.32817	0.33280
C2'	0.18916	0.40571	0.34840
C3'	0.21557	0.48722	0.42408
H3'	0.31234	0.49461	0.47090
C4'	0.11674	0.55786	0.43914
H4'	0.13589	0.62020	0.49793
C5'	-0.00823	0.54793	0.37938
H5'	-0.08460	0.60373	0.39189
C6'	-0.03408	0.46750	0.30392
H6'	-0.12953	0.46143	0.25658
C7'	0.06424	0.39640	0.28818
H7'	0.04565	0.33417	0.22938
C8'	0.39204	0.09318	0.21470
C9'	0.36903	0.09825	0.13224
H9'	0.34969	0.17657	0.11588
C10'	0.37612	0.00265	0.07281
H10'	0.35684	0.00518	0.00841
C11'	0.41032	-0.09421	0.09588
H11'	0.42130	-0.16750	0.04980
C12'	0.43393	-0.09691	0.17869
H12'	0.46003	-0.17280	0.19606
C13'	0.42253	-0.00393	0.23931
H13'	0.43293	-0.00771	0.30347
C14'	0.58923	0.20617	0.38789
C15'	0.56792	0.15672	0.45527
H15'	0.45829	0.14542	0.47705
C16'	0.68732	0.11791	0.48934
H16'	0.67622	0.07649	0.54100
C17'	0.82252	0.12829	0.45594
H17'	0.91370	0.09554	0.48204
C18'	0.84157	0.17908	0.38899
H18'	0.94753	0.18501	0.36372
C19'	0.72424	0.21977	0.35458
H19'	0.73669	0.26103	0.30315

References

1. Penev, K.I.; Bedada, T.; Ray, A.K.; Mequanint, K. Computational studies of 4-nitrophenyl- and 2-benzothiazolyl-substituted formazans and tetrazolium salts. *Chem. Phys.* **2020**, *535*, 110790. https://doi.org/10.1016/j.chemphys.2020.110790

2. Creanga, D.; Nadejde, C. Molecular modelling and spectral investigation of some triphenyltetrazolium chloride derivatives. *Chem. Pap.* **2014**, *68*, 260–271 https://doi.org/10.2478/s11696-013-0429-2

3. Ferjani, H.; Bechaieb, R.; El-Fattah, W.A.; Fettouhi, M. Broad-band luminescence involving fluconazole antifungal drug in a lead-free bismuth iodide perovskite: Combined experimental and computational insights. *Spectrochim. Acta, Part A* **2020**, *237*, 118354. https://doi.org/10.1016/j.saa.2020.118354

4. Ferjani, H.; Bechaieb, R.; Dege, N.; El-Fattah, W.A.; Elamin, N.Y.; Frigui, W. Stabilization of supramolecular network of fluconazole drug polyiodide: Synthesis, computational and spectroscopic studies. *J. Mol. Struct.* **2022**, *1263*, 133192. https://doi.org/10.1016/j.molstruc.2022.133192

5. Ferjani, H.; Bechaieb, R.; Alshammari, M.; Lemine, O.M.; Dege, N. New Organic–Inorganic Salt Based on Fluconazole Drug: TD-DFT Benchmark and Computational Insights into Halogen Substitution. *Int. J. Mol. Sci.* **2022**, *23*, 8765. https://doi.org/10.3390/ijms23158765

6. Gümüş, H.P.; Tamer, Ö.; Avcı, D.; Atalay, Y.; Quantum chemical calculations on the geometrical, conformational, spectroscopic and nonlinear optical parameters of 5-(2-chloroethyl)-2,4-dichloro-6-methylpyrimidine. *Spectrochim. Acta, Part A* **2014**, *129*, 219–226. https://doi.org/10.1016/j.saa.2014.03.031

7. Umemoto, K. Reduction Mechanism of 2,3,5-Triphenyltetrazolium Chloride and 1,3,5-Triphenylformazan. *Bull. Chem. Soc. Jpn.* **1985**, *58*, 2051–2055. https://doi.org/10.1246/bcsj.58.2051
